# Multi-Objective Airflow Distribution Design in Mine Ventilation Systems Based on Sensitivity Screening and an Improved Multi-Objective Sparrow Search Algorithm

**DOI:** 10.3390/biomimetics11080544

**Published:** 2026-08-03

**Authors:** Fengliang Wu, Jianan Gao

**Affiliations:** College of Safety Science and Engineering, Xi’an University of Science and Technology, Xi’an 710054, China; wufl@xust.edu.cn

**Keywords:** biomimetic optimization, improved multi-objective sparrow search algorithm, airflow distribution design, mine ventilation systems, sensitivity screening, multi-objective optimization, Pareto decision support

## Abstract

This study proposes a biomimetic multi-objective optimization framework for airflow distribution design in mine ventilation systems by integrating sensitivity screening with an improved multi-objective sparrow search algorithm (IMOSSA). The design problem is formulated with network-balance, critical branch airflow, fan-boundary, and adjustable-resistance constraints, while theoretical ventilation air power and pressure-drop disturbance are minimized as two conflicting objectives. Resistance-perturbation sensitivity analysis is used to identify high-impact adjustable branches and construct branch-specific search bounds, thereby forming a compact and physically feasible decision domain. Inspired by the foraging and vigilance behaviors of sparrow populations, IMOSSA is employed as a swarm-intelligence Pareto-search engine and integrates three strategies: chaotic opposition-based elite initialization to enhance initial population diversity, density-penalized external-archive guidance to maintain Pareto-front diversity, and stagnation-triggered differential–Cauchy perturbation to improve late-stage escape capability. ZDT and DTLZ benchmark functions verify the computational reliability of IMOSSA; in particular, on the multimodal ZDT4 function, IMOSSA achieves GD, IGD, and HV values of 0.0096, 0.0195, and 0.8479, respectively, indicating strong robustness in complex Pareto-front search. A mine ventilation network case further validates the engineering applicability of the proposed framework. For 13 adjustable branches, the compromise solution reduces model-computed ventilation air power from 313.61 kW to 281.11 kW, corresponding to a reduction of 10.36%, with a pressure-drop deviation of 354.15 Pa; the energy-priority solution further reduces the power to 256.91 kW, corresponding to a reduction of 18.08%. The results show that the proposed biomimetic multi-objective optimization framework can provide computable and interpretable Pareto decision support for airflow distribution design in mine ventilation systems.

## 1. Introduction

Mine ventilation systems are fundamental engineering systems for underground mine safety, air-quality management, thermal and humidity control, and disaster prevention. Their design quality directly affects underground airflow distribution, pollutant dilution, environmental regulation, and the operating load of main fans [1,2]. As mining depth increases, roadway networks expand, and multiple fans operate jointly, mine ventilation systems increasingly exhibit the characteristics of coupled networked engineering systems, in which branches, nodes, fan boundaries, regulating facilities, and air-demand constraints interact with each other. Conventional design procedures based on empirical checking and single-condition calculations are therefore insufficient to simultaneously ensure air-supply reliability, system economy, and comparability among candidate designs [3]. Recent studies have extended mine ventilation research from steady-state network analysis to ventilation-on-demand (VOD), intelligent regulation, parameter identification, and optimization design. Unlike VOD, which focuses on dynamic air-supply regulation during operation, design-stage ventilation optimization aims to generate airflow distribution schemes that satisfy airflow requirements, reduce theoretical ventilation air power, and control adjustment disturbance under a prescribed network topology, fan-boundary conditions, and demand constraints [4].

Mine ventilation network optimization is typically based on steady-state network analysis, in which nodal airflow balance, loop pressure balance, branch resistance laws, and fan-boundary conditions are used to compute airflow, pressure, and air power distributions under different system configurations [5,6,7,8]. Previous studies have addressed complex ventilation network topology representation, feature-graph construction, airflow reconstruction under limited monitoring, multi-fan network solving, branch sensitivity analysis, friction-factor prediction, resistance-parameter identification, and ventilation-resistance calibration [9,10]. In parallel, genetic algorithms, particle swarm optimization, swarm-intelligence methods, mathematical programming, and mixed-integer programming have been applied to ventilation air power reduction, airflow distribution improvement, and regulator configuration [3,11,12,13]. These studies provide important foundations for ventilation-system analysis and optimization; however, many of them focus on state calculation under given resistances, operational regulation, single-objective optimum search, or local adjustment design. Insufficient attention has been paid to how multiple comparable airflow distribution schemes can be generated at the design stage using a limited number of adjustable branch resistances [14].

Data-driven and intelligent methods have also been widely introduced into mine ventilation research. Machine learning, deep learning, Internet of Things (IoT) sensing, and multi-source data fusion have been used for airflow-parameter prediction, ventilation fault diagnosis, and operating-state identification [15,16,17]. Deep reinforcement learning, neuro-fuzzy models, and hybrid intelligent models have further improved ventilation-parameter inversion, risk prediction, and rapid evaluation [18,19]. These methods are effective for learning nonlinear relationships among airflow, pressure, resistance, and environmental variables from monitoring data. Nevertheless, design-stage airflow distribution optimization is not merely a prediction task for a given operating condition; rather, it requires feasible-scheme search and multi-objective trade-off analysis over adjustable-resistance variables under key-branch airflow constraints and fan-boundary conditions [20]. The problem is therefore more appropriately formulated as a constrained multi-objective optimization problem that coordinates theoretical ventilation air power, resistance-adjustment magnitude, and engineering implementability.

Multi-objective evolutionary algorithms, such as NSGA-II, MOPSO, MOEA/D, SPEA2, and multi-objective swarm-intelligence algorithms, do not require analytical gradients and are suitable for nonconvex, strongly coupled, and constrained engineering optimization problems [21,22]. For mine ventilation system design, a Pareto solution set is not only an algorithmic output but also a decision-support representation that provides designers with alternatives reflecting different trade-offs among air power level, resistance disturbance, and airflow distribution performance [23]. However, three gaps remain in existing studies. First, the boundary between design-stage airflow distribution design and operation-stage VOD or online airflow regulation is often blurred. Second, some studies aggregate airflow deviation, energy consumption, number of regulators, and adjustment magnitude into a single fitness value, weakening the physical meaning of individual objectives and the interpretability of Pareto solutions. Third, adjustable-branch selection and resistance bounds are often specified empirically, with limited integration of network airflow-response sensitivity; this may introduce low-contribution variables and increase the optimization dimension.

To address these gaps, this study proposes a sensitivity screening and IMOSSA-based biomimetic multi-objective optimization method for airflow distribution design in mine ventilation systems. Adjustable branch resistances are used as system design variables, key-branch airflow and fan-boundary airflow are imposed as constraints, and theoretical ventilation air power and adjustable-branch pressure-drop deviation are minimized as two conflicting objectives. A resistance-perturbation sensitivity analysis is introduced to identify high-impact adjustable branches and derive branch-specific search bounds based on sensitivity attenuation, thereby constructing a compact and physically feasible decision domain. Inspired by the foraging and vigilance behaviors of sparrow populations, the improved multi-objective sparrow search algorithm (IMOSSA) is employed as a swarm-intelligence Pareto-search engine. Chaotic opposition-based elite initialization, density-penalized external-archive guidance, and stagnation-triggered differential–Cauchy perturbation are embedded to enhance initial design-space coverage, Pareto-scheme diversity, and late-stage escape capability. Standard multi-objective benchmark functions and a mine ventilation network case are used to evaluate the computational reliability, complex Pareto-front search performance, and engineering applicability of the proposed method. From a biomimetics perspective, this study demonstrates how a sparrow-inspired search mechanism can be adapted to solve a constrained airflow distribution design problem in a coupled engineering ventilation system.

## 2. Bi-Objective Model for Airflow Distribution Design in Mine Ventilation Systems

### 2.1. Sensitivity-Based Screening of Adjustable Branches

A key step in airflow distribution design for mine ventilation systems is to determine which branch resistances should be adjustable. Treating all branch resistances as decision variables can enlarge the search space, but it also substantially increases the problem dimension and may introduce low-contribution branches that provide limited improvement in demand-critical airflow while disturbing the overall network distribution [23]. From a systems perspective, adjustable branches can be regarded as controllable components that influence the overall airflow distribution. Therefore, this study develops a sensitivity-based branch screening method using branch-resistance-perturbation responses, aiming to identify adjustable branches with strong control ability over demand-critical airflow and limited influence on non-target regions.

Let the mine ventilation network contain $N_{b}$ branches. The initial resistance vector is denoted by $R^{0}=(R_{1}^{0},R_{2}^{0},\cdots ,R_{N_{b}}^{0}{)}^{T}$, and the airflow vector obtained by network solving is denoted by q(R). The set of demand-critical branches is denoted by $\Omega_{d}$, and the non-target observation set is denoted by $\Omega_{o}$. For a candidate branch j, proportional perturbations +δ and −δ are applied to its initial resistance to obtain $R_{j}^{+}$ and $R_{j}^{-}$. In this study, δ=0.01, corresponding to a ±1% perturbation of the initial branch resistance. The airflow-response sensitivities of branch j to the demand-critical and non-target branches are defined as follows:

(1) $$S_{j}^{d}=∥\frac{|q_{\Omega_{d}}(R_{j}^{+})|-|q_{\Omega_{d}}(R_{j}^{-})|}{2\delta R_{j}^{0}}{∥}_{2},S_{j}^{o}=∥\frac{|q_{\Omega_{o}}(R_{j}^{+})|-|q_{\Omega_{o}}(R_{j}^{-})|}{2\delta R_{j}^{0}}{∥}_{2}$$

Here, $S_{j}^{d}$ measures the target-control capability of branch j, whereas $S_{j}^{o}$ measures its disturbance effect on non-target branches. To evaluate the control benefit and disturbance cost simultaneously, the comprehensive regulation coefficient is defined as:

(2) $$\Gamma_{j}=\frac{S_{j}^{d}}{S_{j}^{o}+\varepsilon }$$

where ε is a small positive constant used to avoid division by zero. A larger $\Gamma_{j}$ indicates a stronger response to demand-critical airflow and weaker disturbance to non-target regions. The non-target impact cost is further defined as:

(3) $$I_{j}=\frac{S_{j}^{o}+\varepsilon }{S_{j}^{d}+\varepsilon }$$

Using $I_{j}$ as the edge weight, the minimum-impact tree is constructed as

(4) $$T^{*}=arg\;\underset{T\in T}{min}\sum_{j\in T}I_{j}$$

where T denotes the set of candidate trees satisfying network connectivity, branch adjustability, and engineering installation constraints, and $T^{*}$ is the resulting minimum-impact tree.

In implementation, the original ventilation network is converted into a candidate undirected weighted graph $G_{reg}=(V,E_{reg},w)$, where $E_{reg}$ contains only branches on which resistance adjustment is practically feasible. Fan branches, non-constructible branches, and branches that should not be regulated for safety reasons are excluded from the candidate set, and $I_{j}$ is assigned as the edge weight. In this study, the Kruskal algorithm is used to construct the minimum-impact tree $T^{*}$. The branches in $T^{*}$ are ranked in descending order of $\Gamma_{j}$, and the first K branches are selected to form the adjustable branch set $\Omega_{reg}$. The minimum-impact tree is used only as a screening structure to avoid excessive concentration of adjustable branches in a local region; no cotree-based selection or graph-compensation operation is involved. This procedure determines which branch resistances enter the optimization model and reduces the decision dimension from the full engineering-adjustable candidate set to K high-contribution branches, thereby constituting the variable-screening part of the sensitivity-based decision-domain construction.

### 2.2. Determination of Resistance Search Bounds for Adjustable Branches

After the adjustable branch set is determined, suitable resistance bounds must be assigned to each branch. Uniform proportional bounds may restrict effective adjustment for highly sensitive branches while assigning unnecessarily wide search intervals to weakly sensitive branches. Following the sensitivity-attenuation principle used for resistance-bound determination [24], this study constructs branch-specific resistance search bounds according to the attenuation of target-airflow response induced by branch-resistance perturbation.

For an adjustable branch $j\in \Omega_{reg}$, let μ be the resistance-perturbation multiplier. The resistance vector after changing only branch j is denoted by $R_{j}(\mu )$, where the j-th branch resistance is $\mu R_{j}^{0}$ and all other branch resistances remain unchanged. A central-difference scheme is used to estimate the sensitivity of demand-critical airflow around μ:

(5) $$D_{j}(\mu )=\frac{|q_{\Omega_{d}}(R_{j}(\mu +\delta_{\mu }))|-|q_{\Omega_{d}}(R_{j}(\mu -\delta_{\mu }))|}{2\delta_{\mu }R_{j}^{0}},S_{j}(\mu )=∥D_{j}(\mu ){∥}_{2}$$

Here, $\delta_{\mu }$ is the perturbation increment and is set to 0.01 in this study, corresponding to a ±1% local variation relative to the initial branch resistance. This small relative increment maintains the local character of the central-difference estimate while avoiding airflow differences that are excessively small relative to the numerical solution tolerance, consistent with the resistance-perturbation treatment used in related sensitivity-based ventilation studies [24]. In general, as the perturbation magnitude increases, the marginal airflow improvement obtained by further expanding the resistance adjustment range gradually decreases. Let $\varepsilon_{s}$ be the sensitivity-attenuation threshold and let $S_{j}\left(1\right)$ be the initial response. The effective perturbation multipliers in the resistance-increasing and resistance-decreasing directions are then given by

(6) $$\mu_{j}^{+}=min\{\mu \in M^{+}|S_{j}(\mu )\leq \varepsilon_{s}S_{j}(1)\},\mu_{j}^{-}=max\{\mu \in M^{-}|S_{j}(\mu )\leq \varepsilon_{s}S_{j}(1)\}$$

Here, $M^{+}$ and $M^{-}$ are the candidate multiplier sets for increasing and decreasing resistance, respectively. If the attenuation threshold is not reached, the engineering boundary in the corresponding direction is used. The resistance interval for branch j is therefore determined as

(7) $$R_{j}^{L}=max(R_{j,min},\mu_{j}^{-}R_{j}^{0}),R_{j}^{U}=min(R_{j,max},\mu_{j}^{+}R_{j}^{0})$$

In this expression, $R_{j}^{L}$ and $R_{j}^{U}$ are the lower and upper bounds of the adjustable resistance, whereas $R_{j,min}$ and $R_{j,max}$ are determined by roadway conditions, regulator capacity, and engineering experience. If only resistance-increasing devices such as regulators, air doors, or air windows are considered, $R_{j}^{L}$ may be set to $R_{j}^{0}$. During interval generation, $M^{+}$ and $M^{-}$ are bound by feasible engineering ranges, and $\varepsilon_{s}$ is used only to compress the search interval within this feasible domain. Multipliers that cause airflow reversal, network-solution failure, or violation of non-adjustability constraints are excluded. This rule maintains physical implementability and prevents low-contribution branches from generating excessively wide search ranges.

### 2.3. Bi-Objective Airflow Distribution Design Model

After branch screening and bound determination, the adjustable branch resistances are used as system design variables to construct a bi-objective optimization model for airflow distribution design. Let $\Omega_{reg}=\left\{j_{1},j_{2},\cdots ,j_{K}\right\}$ and $x=\left(R_{j_{1}},R_{j_{2}},\cdots ,R_{j_{K}}\right)$ denote the adjustable branch set and the corresponding decision vector. For any candidate solution x, the vector is mapped to the full-network resistance vector, and the ventilation network solver is called to obtain branch airflow $q_{j}(x)$, branch pressure drop $h_{j}(x)$, fan-boundary airflow $Q_{f}(x)$, and equivalent fan pressure $H_{f}(x)$. Demand-critical branch airflow is treated as a constraint rather than an objective, and the number of adjustable branches is analyzed separately through branch-number sensitivity tests.

The constraints include resistance bounds, nodal airflow balance, loop pressure balance, demand-critical branch airflow constraints, and fan-boundary airflow constraints [14]:

(8) $$R_{j}^{L}\leq R_{j}(x)\leq R_{j}^{U},j\in \Omega_{reg}$$

(9) $$\sum_{j\in \Omega_{n}^{in}}q_{j}(x)=\sum_{j\in \Omega_{n}^{out}}q_{j}(x),n=1,2,\cdots ,N_{n}$$

(10) $$\sum_{j\in L_{l}}\sigma_{lj}R_{j}(x)q_{j}(x)|q_{j}(x)|-\sum_{f\in L_{l}}\sigma_{lf}H_{f}(x)-H_{l}^{n}=0,l=1,2,\cdots ,N_{l}$$

(11) $$Q_{i}^{L}\leq |q_{i}(x)|\leq Q_{i}^{U},i\in \Omega_{d};Q_{f}^{L}\leq |Q_{f}(x)|\leq Q_{f}^{U},f\in \Omega_{f}$$

According to the commonly used ventilation air power calculation in mine ventilation optimization [3], the first objective is expressed as:

(12) $$f_{1}(x)=\sum_{f=1}^{N_{f}}\frac{|H_{f}(x)Q_{f}(x)|}{1000}$$

Here, $N_{f}$ is the number of fan-boundary branches, and $f_{1}\left(x\right)$ is measured in kW. Because the fan-efficiency curve is not introduced, $f_{1}\left(x\right)$ represents the theoretical air power associated with the product of equivalent fan pressure and airflow, rather than the actual electrical energy consumption of the fan system.

To quantify the disturbance caused by resistance adjustment and airflow redistribution, this study defines the second objective as the pressure-drop variation in adjustable branches. If the initial pressure drop of branch j is $h_{j}^{0}=R_{j}^{0}|q_{j}^{0}{|}^{2}$ and the optimized pressure drop is $h_{j}(x)=R_{j}(x)|q_{j}(x){|}^{2}$, then

(13) $$f_{2}(x)=\sum_{j\in \Omega_{reg}}|h_{j}(x)-h_{j}^{0}|=\sum_{j\in \Omega_{reg}}|R_{j}(x)|q_{j}(x){|}^{2}-R_{j}^{0}|q_{j}^{0}{|}^{2}|$$

The unit of $f_{2}\left(x\right)$ is Pa. This objective quantifies the disturbance imposed on the initial ventilation state by resistance adjustment and airflow redistribution. Accordingly, the complete bi-objective model established in this study can be written as

(14) $$\underset{x}{\min}\;F\left(x\right)=\left[f_{1}\left(x\right),f_{2}\left(x\right)\right],\;\mathrm{subject}\;\mathrm{to}\;\left(8\right)–(11)$$

Here, $N_{n}$ and $N_{l}$ denote the numbers of nodes and independent loops, respectively; $\Omega_{d}$ and $\Omega_{f}$ are the demand-critical branch set and fan-boundary branch set; $\Omega_{n}^{in}$ and $\Omega_{n}^{out}$ are the inflow and outflow branch sets of node n; $L_{l}$ is the l-th independent loop; $\sigma_{lj}$ and $\sigma_{lf}$ are direction-consistency coefficients; and $H_{l}^{n}$ denotes natural ventilation pressure. If natural ventilation pressure is not considered, $H_{l}^{n}$ is set to zero. Each optimization individual therefore corresponds to a complete airflow distribution design scheme represented by adjustable branch settings. After network solving, theoretical ventilation air power, pressure-drop disturbance, and constraint satisfaction can be evaluated. IMOSSA is then used to search for Pareto non-dominated schemes that balance low air power and low adjustment disturbance, providing alternatives for design-stage airflow distribution design and multi-scheme comparison.

## 3. Improved Multi-Objective Sparrow Search Algorithm for Pareto Design Search

In the proposed system-oriented optimization framework, IMOSSA is used as the computational engine for searching Pareto design alternatives in the airflow distribution design problem. Its role is not only to improve numerical optimization performance, but also to provide a diverse set of feasible schemes under multiple system constraints.

### 3.1. Multi-Objective Sparrow Search Algorithm

The sparrow search algorithm (SSA) simulates the discoverer, follower, and vigilant behaviors of sparrow populations to achieve global exploration and local exploitation [24,25]. The airflow distribution design problem in a mine ventilation system involves two conflicting objectives, namely theoretical ventilation air power and pressure-drop variation, and is constrained by airflow bounds, resistance ranges, and network balance. Therefore, this study extends SSA by introducing non-dominated sorting, crowding distance, and an external archive, thereby forming the multi-objective sparrow search algorithm (MOSSA).

Let N be the population size, D the decision dimension, and $x_{i}^{t}=(x_{i,1}^{t},x_{i,2}^{t},\cdots ,x_{i,D}^{t})$ the i-th individual at iteration t. Its objective vector is $F(x_{i}^{t})=(f_{1}(x_{i}^{t}),f_{2}(x_{i}^{t}),\cdots ,f_{M}(x_{i}^{t})).$

Individual $x_{a}$ is said to dominate $x_{b}$ if it is no worse than $x_{b}$ in all objectives and better in at least one objective. Based on this dominance relation, non-dominated sorting is performed, and crowding distance is used to preserve the distribution uniformity of the Pareto front. For role assignments, individuals are ranked by non-dominated rank $r_{i}$, normalized objective score, and crowding distance. The normalized objective score is defined as:

(15) $$s_{i}=\sum_{m=1}^{M}\frac{f_{m}(x_{i})-\underset{1\leq k\leq N}{min}f_{m}(x_{k})}{\underset{1\leq k\leq N}{max}f_{m}(x_{k})-\underset{1\leq k\leq N}{min}f_{m}(x_{k})+\varepsilon }$$

Individuals with lower non-dominated ranks, smaller objective scores, and larger crowding distances are preferentially selected to balance convergence and diversity. At each iteration, the external archive stores the current non-dominated solutions and provides a guiding individual $g^{t}$. The worst individual in the current population is denoted by $x_{w}^{t}$, and the maximum number of iterations is T. Under a safe state, discoverers are updated as:

(16) $$x_{i}^{t+1}=x_{i}^{t}+(1-exp(\frac{-p}{\alpha T}))(g^{t}-x_{i}^{t})+\sigma_{t}z⊙(u-l)$$

Here, p is the ranking position, α is a random coefficient, z is a standard normal random vector, u and l are the upper and lower bounds of the decision variables, and $\sigma_{t}\;$is a decreasing perturbation coefficient. The negative exponential term keeps the guidance step bounded with respect to the ranking position and iteration scale, thereby avoiding abnormal amplification along the search direction.

Followers perform differentiated searches according to their ranking positions. Poorly ranked individuals move away from the worst individual to expand the search range, whereas better individuals exploit the region around the archive-guided solution:

(17) $$x_{i}^{t+1}=\left\{\begin{array}{cc} x_{i}^{t}+z⊙|x_{i}^{t}-x_{w}^{t}|+\lambda_{t}z⊙(u-l), & p>N/2\\ g^{t}+|x_{i}^{t}-g^{t}|⊙A⊙r, & p\leq N/2 \end{array}\right.$$

where $\lambda_{t}\;$is an iteration-dependent decreasing search scale, which allows broader exploration in the early iterations and reduces excessive displacement during later refinement. Each element of A is independently selected from $\left\{-1,1\right\}\;$with equal probability to provide unbiased positive or negative search directions in each decision dimension, while r is a random vector in ${\left[0,1\right]}^{D}\;$that controls the corresponding step magnitude. Vigilant individuals provide supplementary exploration: inferior individuals re-explore around $g^{t}$, whereas better individuals move away from $x_{w}^{t}\;$to reduce the risk of entering unfavorable regions. After each iteration, parent, offspring, and archive solutions are merged, and environmental selection based on non-dominated sorting and crowding distance is used to generate the next population and update the archive.

### 3.2. Improvement Strategies

Although MOSSA can solve multi-objective optimization problems, the design space of mine ventilation airflow distribution may still suffer from insufficient initial coverage, archive guidance biased toward locally dense regions, and weak late-stage search activity [26]. To improve its suitability for system-oriented Pareto design search, this study embeds three strategies into MOSSA: chaotic opposition-based elite initialization, density-penalized external-archive guidance, and stagnation-triggered differential–Cauchy perturbation. These strategies are introduced to improve the initial coverage of the complex design space, maintain the diversity of Pareto design alternatives, and reduce the risk of being trapped in local feasible regions. Compared with MOSSA, these modifications provide three direct benefits: broader initial coverage of the design space, less biased archive guidance with improved Pareto-set diversity, and stronger late-stage escape from local Pareto regions. Consequently, IMOSSA improves convergence robustness and solution-distribution quality while retaining the basic role-based search structure of MOSSA.

#### 3.2.1. Chaotic Opposition-Based Elite Initialization

The initial population directly affects early convergence and Pareto-front coverage. For system design problems with continuous decision variables and constrained feasible regions, insufficient initial coverage may reduce the diversity of candidate design schemes. To mitigate uneven random initialization in high-dimensional continuous spaces, logistic chaotic mapping and opposition-based learning are combined to generate candidate populations [27]. If $c_{i,d}\;$is the d-th chaotic variable of individual i, then

(18) $$c_{i,d}^{k+1}=4c_{i,d}^{k}(1-c_{i,d}^{k}),x_{i,d}^{c}=l_{d}+c_{i,d}^{K}(u_{d}-l_{d})$$

where K is the number of chaotic iterations, and $l_{d}\;$and $u_{d}$ are the lower and upper bounds of the d-th decision variable, respectively. The opposite solution is constructed as:

(19) $$x_{i,d}^{o}=l_{d}+u_{d}-x_{i,d}^{c}$$

The random, chaotic, and opposite populations are merged and evaluated. Non-dominated sorting and crowding-distance selection are then used to retain N high-quality and well-distributed individuals as the initial population. This strategy improves the initial search coverage without changing the main iterative structure.

#### 3.2.2. Density-Penalized External-Archive Guidance

The external archive stores non-dominated solutions and provides search directions for population updating. In a system design context, the archive should not only guide convergence but also maintain diverse Pareto alternatives for subsequent scheme comparison. If the guiding solution is selected only according to crowding distance, the population may repeatedly follow locally dense archive regions, weakening the overall distribution of the Pareto front. To encourage exploration of sparse regions, a density-penalized external-archive guidance mechanism is introduced [28].

Before density calculation, the objective values in the archive are range-normalized to eliminate scale differences among objectives. The local density of the a-th archive solution is defined as:

(20) $$\rho_{a}=\frac{1}{\underset{b\in A,b\neq a}{min}∥F_{a}-F_{b}{∥}_{2}+\varepsilon }$$

A larger $\rho_{a}\;$indicates a denser neighborhood. To increase the probability that sparse-region solutions are selected as guiding individuals, the density-penalized selection probability is defined as:

(21) $$P_{a}=\frac{CD_{a}/(\rho_{a}+\varepsilon )}{\sum_{b\in A}CD_{b}/(\rho_{b}+\varepsilon )}$$

The guiding solution $g^{t}\;$is randomly selected from the archive according to $P_{a}$. In implementation, infinite boundary crowding distances are truncated to the maximum finite crowding distance in the archive and then normalized to $\left[0,1\right]$. If the archive contains fewer than three solutions or all finite crowding distances are identical, uniform selection is used to avoid non-normalizable probabilities or excessive amplification of boundary solutions.

#### 3.2.3. Stagnation-Triggered Adaptive Differential–Cauchy Perturbation

In late iterations, the population may concentrate around a local Pareto region, slowing archive improvement. To enhance escape ability, a differential–Cauchy perturbation strategy is triggered only when archive progress stagnates [29]. Let $G\left(t\right)\;$be the archive-progress indicator at iteration t. For benchmark tests, $G\left(t\right)\;$can be IGD; for engineering problems, it can be the minimum normalized aggregate objective score in the archive. Following the stagnation-detection principle based on the absence of effective improvement over consecutive generations [30], if the following condition holds over a window of W iterations, the algorithm is regarded as stagnated:

(22) $$G(t-W)-G(t)\leq \eta max\left(1,|G(t-W)|\right)$$

where W is the detection window and η is the improvement tolerance. Once stagnation is detected, the DE/rand/1 mutation and binomial crossover operators are introduced [31]. Specifically, $x_{r1}$, $x_{r2}$, and $x_{r3}\;$are randomly selected from the combined population and archive to generate a differential mutant:

(23) $$v=x_{r1}+F_{de}(x_{r2}-x_{r3})$$

The mutant vector is then combined with the current individual through binomial crossover:

(24) $$u_{d}=\left\{\begin{array}{cc} v_{d}, & {rand}_{d}\leq CR\;\mathrm{or}\;d=d_{rand}\\ x_{i,d}^{t}, & otherwise \end{array}\right.$$

where $F_{de}\;$is the differential scaling factor, CR is the crossover probability, ${rand}_{d}\;$is an independently generated random number for the d-th dimension, and $d_{rand}\;$ensures that at least one component is inherited from the mutant vector:

(25) $$x_{i}^{t+1}=x_{i}^{t}+\beta_{t}c⊙(u-l)+\gamma (g^{t}-x_{i}^{t})⊙r$$

where c is a Cauchy random vector, $\beta_{t}$ is a decreasing perturbation coefficient, and γ is a guidance weight. Differential perturbation generates new directions from population differences, while the heavy-tailed Cauchy perturbation increases the probability of escaping local regions. The guidance term keeps the perturbation near the current non-dominated search area.

### 3.3. IMOSSA Procedure and Computational Complexity

**Figure 1:**
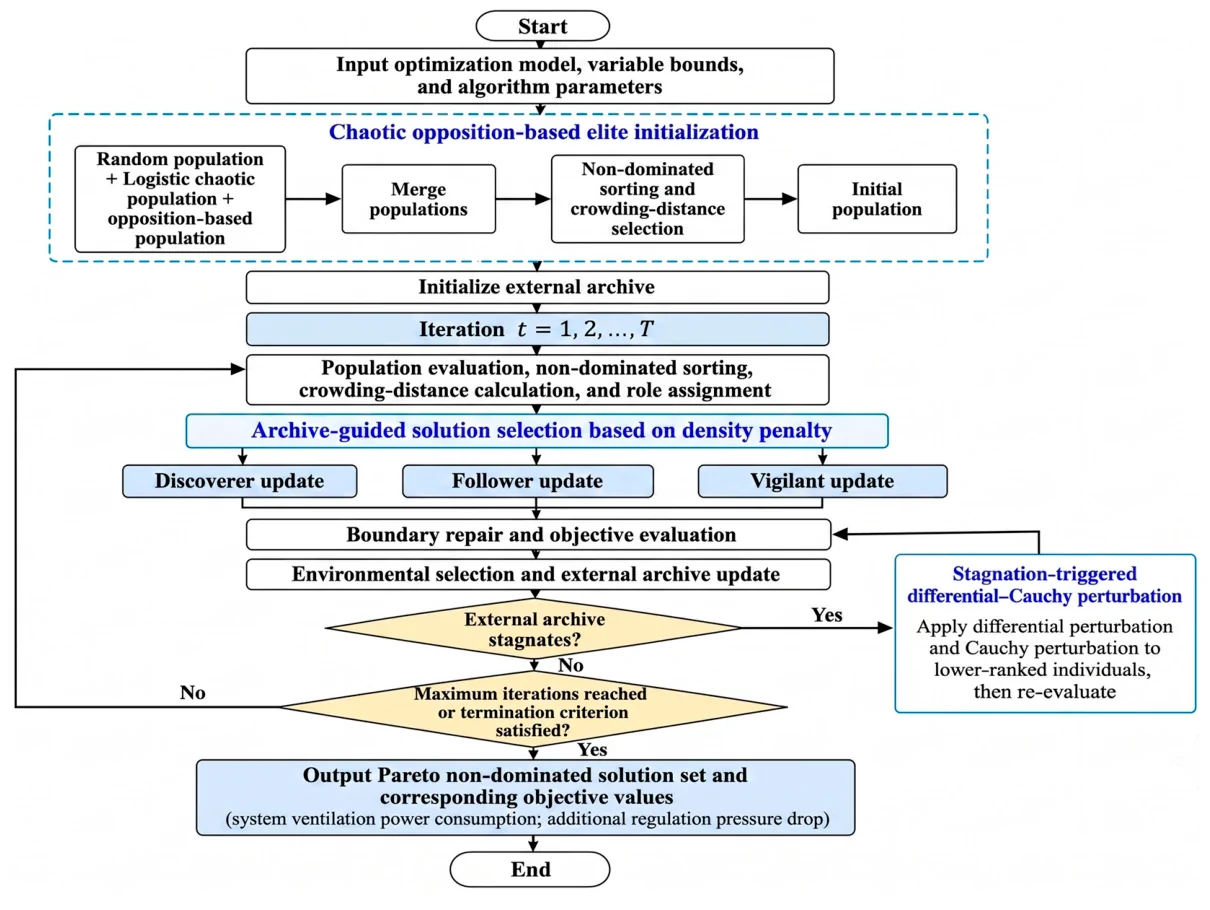
Flowchart of the proposed IMOSSA. Blue-highlighted elements distinguish the main algorithmic modules, while yellow diamonds denote decision nodes; the colors are used only for visual grouping.

The complete IMOSSA procedure is illustrated in Figure 1. The algorithm first inputs the multi-objective airflow distribution design model, variable bounds, and control parameters, including population size N, maximum iterations T, archive capacity $N_{A}$, discoverer ratio PD, vigilant ratio SD, safety threshold ST, and perturbation parameters. Random, logistic chaotic, and opposite populations are generated and merged. Objective evaluation, non-dominated sorting, and crowding-distance selection are then used to form the initial population, and non-dominated individuals are extracted to initialize the external archive.

During iteration, objective values are normalized, individuals are sorted and assigned roles, and the guiding solution $g^{t}\;$is selected from the archive according to the density-penalized probability. Under this guidance, discoverers, followers, and vigilant individuals perform their respective updates. Updated individuals are repaired to the variable bounds, evaluated by the objective functions and constraint violation, and then merged with parent and archive members for environmental selection and archive updating.

If the archive shows insufficient progress over consecutive generations, differential–Cauchy perturbation is applied only to lower-ranked individuals, followed by boundary repair and re-evaluation. When the maximum number of iterations or another termination condition is reached, the external archive contains the Pareto solution set. In the airflow distribution design problem, each non-dominated individual corresponds to an adjustable branch setting scheme, and its objective values represent theoretical ventilation air power and pressure-drop variation.

The main computational cost of IMOSSA arises from solution evaluation, non-dominated sorting, and archive maintenance. For population size N, decision dimension D, objective number M, maximum iterations T, and archive capacity $N_{A}$, the complexity of one role-update stage is approximately O(ND), while the worst-case complexity of non-dominated sorting and archive updating is $O(M(N+N_{A}{)}^{2})$. In mine ventilation applications, each generation also calls the network solver; therefore, the actual runtime is mainly affected by network size, number of function evaluations, and archive capacity.

The proposed method is intended for design-stage and offline Pareto scheme generation rather than high-frequency real-time airflow control. In practical application, sensitivity screening is first used to reduce the number of adjustable branches, after which IMOSSA searches the reduced decision domain and generates a set of feasible Pareto schemes. Engineers can then select energy-priority, low-disturbance, or compromise schemes according to design and retrofit requirements. Under the computational settings used in this study, the reported runtime is at the minute scale, which is acceptable for ventilation system planning, regulator configuration, and scheme comparison.

## 4. Experiments and Analysis

### 4.1. Benchmark Verification of the Computational Reliability of IMOSSA

Before applying IMOSSA to the mine ventilation system case, benchmark tests were conducted to verify the computational reliability, convergence behavior, and distribution capability of the optimizer. The test functions include ZDT1, ZDT2, ZDT3, ZDT4, and the three-objective DTLZ1, which represent convex fronts, nonconvex fronts, discontinuous fronts, multimodal search spaces, and complex three-objective fronts, respectively. The compared algorithms include NSGA-II, MOEA/D, SPEA2, MODE, MOSSA, and IMOSSA. All experiments were implemented in MATLAB R2023a. Four metrics were adopted: GD, IGD, HV, and Spacing. Smaller GD and IGD values indicate closer approximation to the true Pareto front, a larger HV indicates better convergence and coverage, and a smaller Spacing value indicates a more uniform distribution.

**Figure 2:**
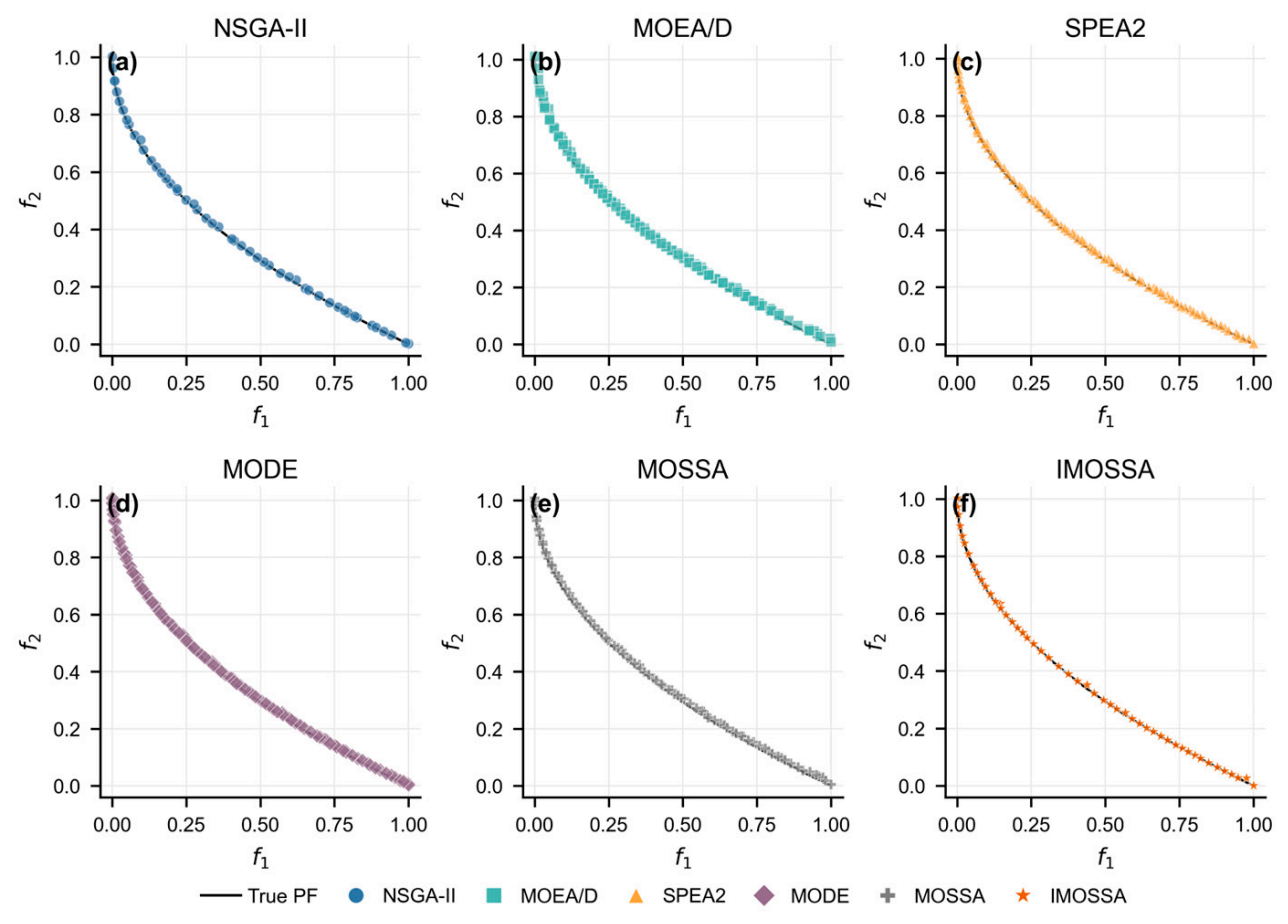
Pareto-front comparison on ZDT1: (**a**) NSGA-II; (**b**) MOEA/D; (**c**) SPEA2; (**d**) MODE; (**e**) MOSSA; (**f**) IMOSSA. The black lines denote the true Pareto front, and the colored markers denote the solutions generated by the corresponding algorithms.

**Figure 3:**
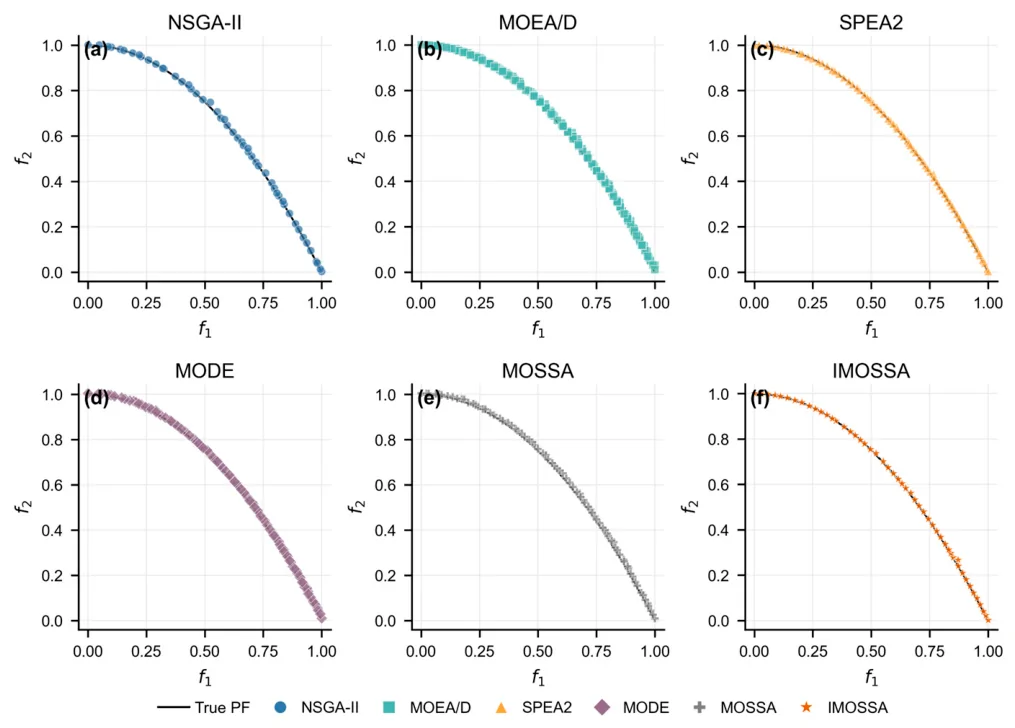
Pareto-front comparison on ZDT2: (**a**) NSGA-II; (**b**) MOEA/D; (**c**) SPEA2; (**d**) MODE; (**e**) MOSSA; (**f**) IMOSSA.

Figure 2 and Figure 3 show the Pareto fronts obtained on ZDT1 and ZDT2, and Table 1 reports the corresponding performance metrics. Both functions are used to examine the basic search ability of the algorithms on continuous fronts. IMOSSA closely approximates the true fronts and maintains stable coverage at both the endpoints and the middle-front region. Its GD and HV values are $0.0033\pm 6.61\times 10^{-4}$ and $0.8635\pm 6.45\times 10^{-4}$ on ZDT1, and $0.0024\pm 6.55\times 10^{-4}$ and $0.5309\pm 7.21\times 10^{-4}$ on ZDT2, respectively. These results indicate that chaotic opposition-based initialization and density-penalized archive guidance help improve initial coverage and Pareto-front preservation.

**Figure 4:**
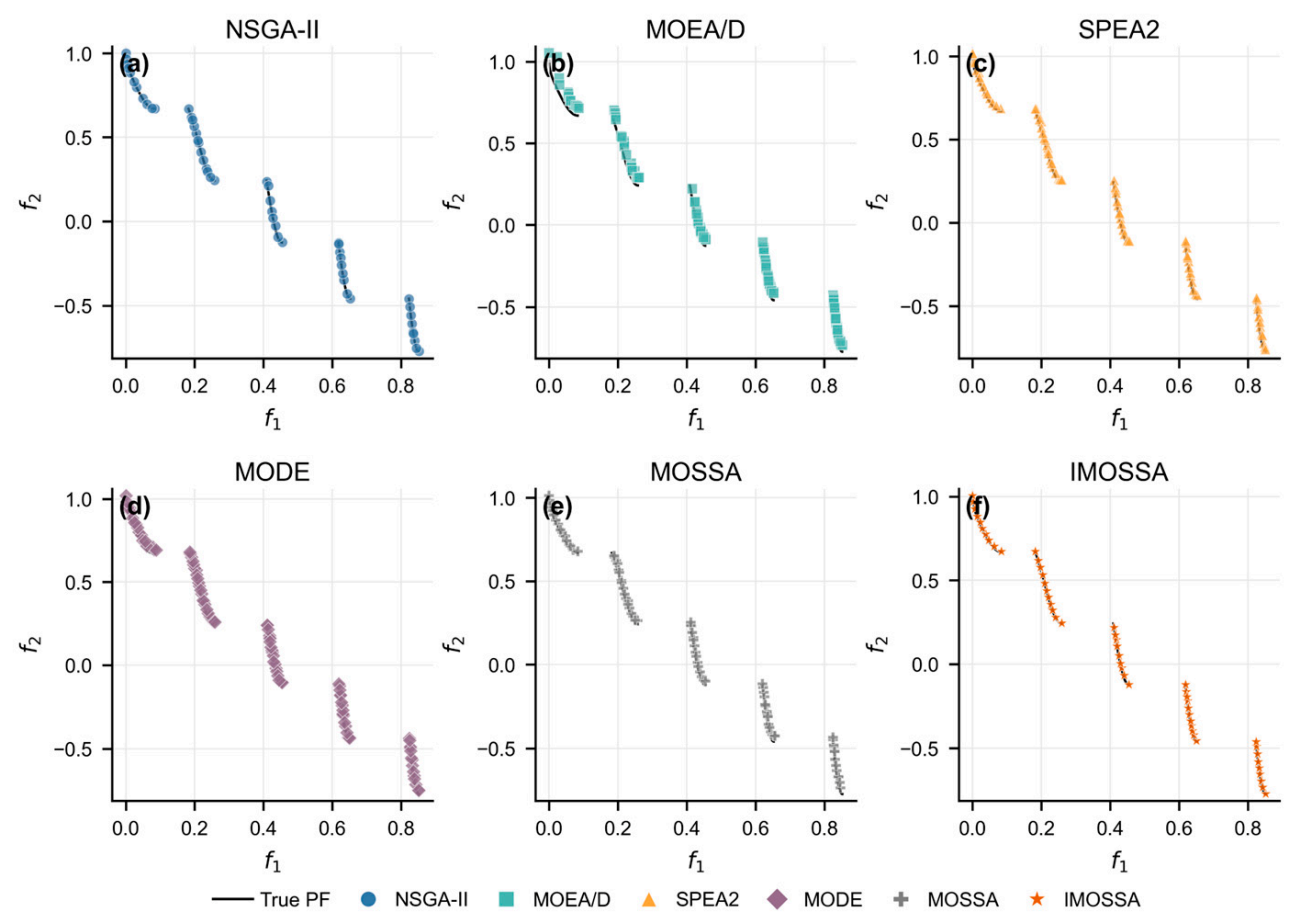
Pareto-front comparison on ZDT3: (**a**) NSGA-II; (**b**) MOEA/D; (**c**) SPEA2; (**d**) MODE; (**e**) MOSSA; (**f**) IMOSSA.

Figure 4 presents the results on the discontinuous ZDT3 front. Since the true Pareto front consists of several separated segments, an effective algorithm should not only approximate these segments but also avoid generating solutions in disconnected invalid regions. IMOSSA identifies multiple separated front segments and maintains relatively uniform distributions within them. Its Spacing value is 0.0085±0.0012, which is better than those of the compared algorithms, suggesting that density-penalized archive guidance reduces local over-concentration. However, IMOSSA is not optimal in terms of IGD on ZDT3, indicating that its global coverage precision on discontinuous fronts could still be improved.

**Figure 5:**
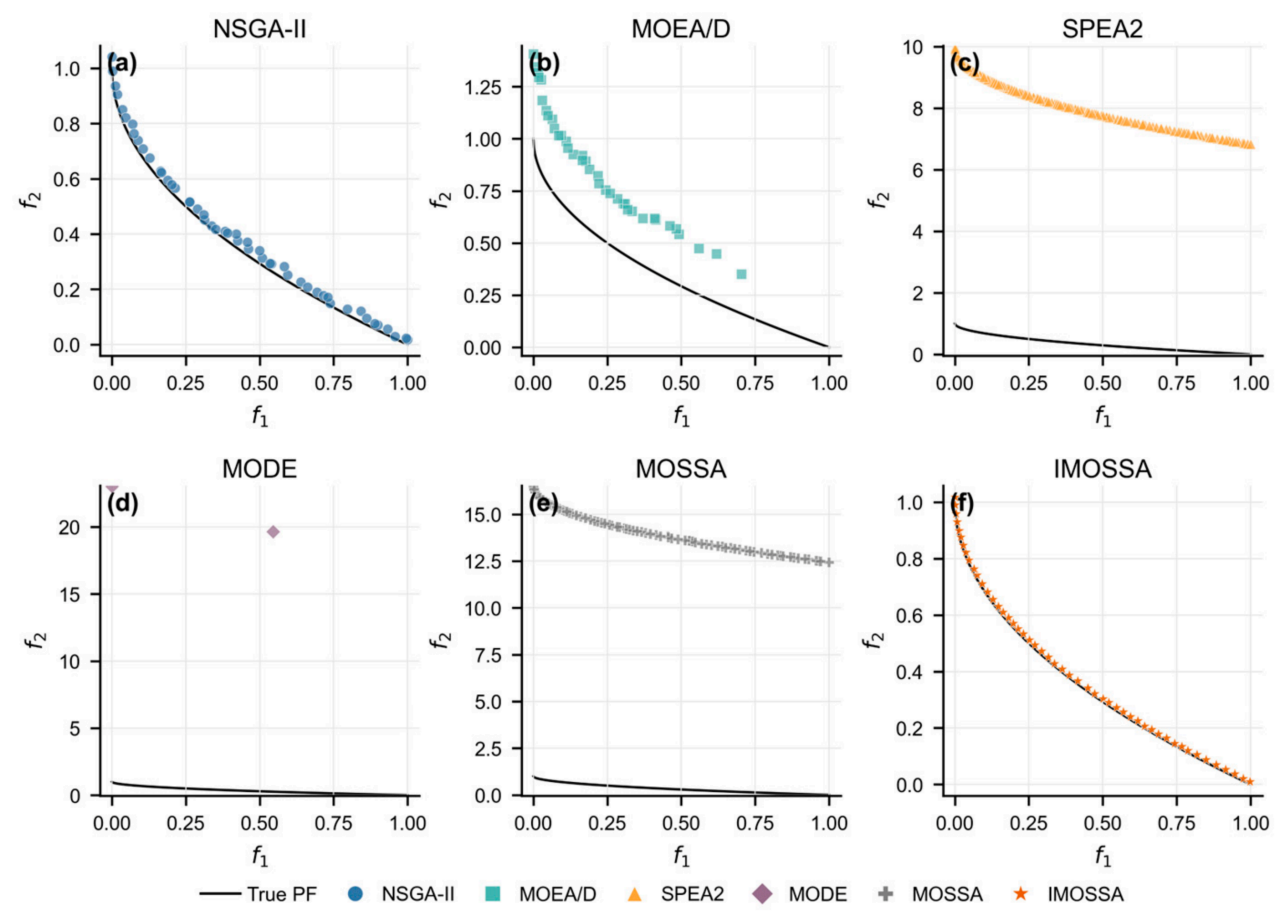
Pareto-front comparison on ZDT4: (**a**) NSGA-II; (**b**) MOEA/D; (**c**) SPEA2; (**d**) MODE; (**e**) MOSSA; (**f**) IMOSSA.

ZDT4 is a multimodal function with many local Pareto fronts and is therefore useful for examining the ability to escape local regions. As shown in Figure 5, SPEA2, MODE, and MOSSA deviate substantially from the true front, whereas IMOSSA maintains better agreement and continuity. On ZDT4, the GD, IGD, and HV values of IMOSSA are 0.0096±0.0048, 0.0195±0.0216, and 0.8479±0.0190, respectively, clearly outperforming the original MOSSA and most alternatives. This result supports the effectiveness of the stagnation-triggered differential–Cauchy perturbation in enhancing late-stage search activity and helping the population escape local Pareto regions.

**Figure 6:**
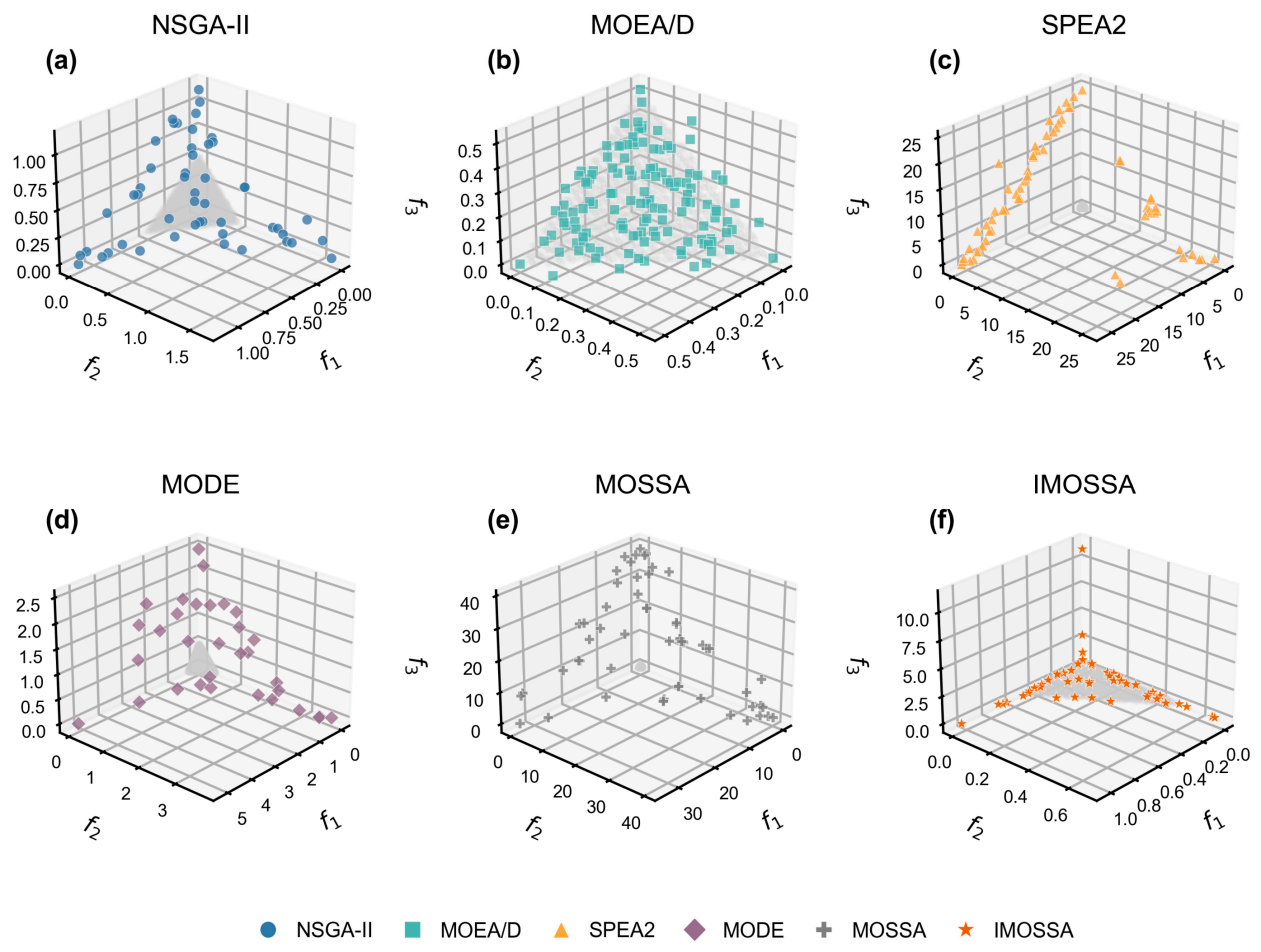
Three-dimensional Pareto-front comparison on DTLZ1: (**a**) NSGA-II; (**b**) MOEA/D; (**c**) SPEA2; (**d**) MODE; (**e**) MOSSA; (**f**) IMOSSA.

**Figure 7:**
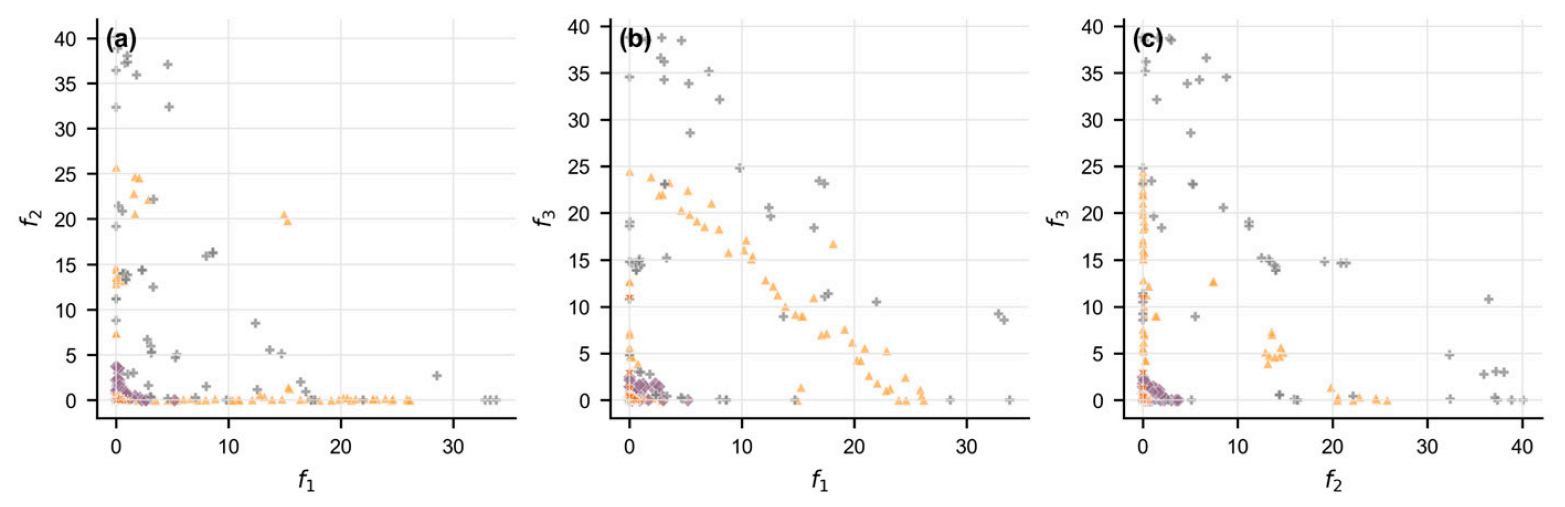
Two-dimensional projections of DTLZ1 Pareto solutions: (**a**) $f_{1}$–$f_{2}$ projection; (**b**) $f_{1}$–$f_{3}$ projection; (**c**) $f_{2}$–$f_{3}$ projection. The marker colors and shapes correspond to the algorithms shown in the legend of Figure 6.

For the three-objective DTLZ1 problem, Figure 6 shows the Pareto solutions in the three-dimensional objective space, and Figure 7 gives their two-dimensional projections. Compared with two-objective problems, the weakened dominance relation in three-objective optimization makes solution dispersion, front deviation, and high-objective residual points more likely. The IMOSSA solutions are generally closer to the low-objective region and show acceptable coverage in the projections, indicating a degree of search stability in a more complex objective space. Nevertheless, Table 1 shows that IMOSSA is not the best algorithm for all DTLZ1 metrics. In particular, the relatively large standard deviation of the Spacing metric indicates noticeable run-to-run variability in solution-distribution uniformity, which is consistent with the occasional occurrence of sparsely distributed or high-objective residual solutions in the three-objective search space. Therefore, although IMOSSA remains competitive in terms of IGD and HV, its distribution stability in higher-dimensional objective spaces still requires further improvement.

**Figure 8:**
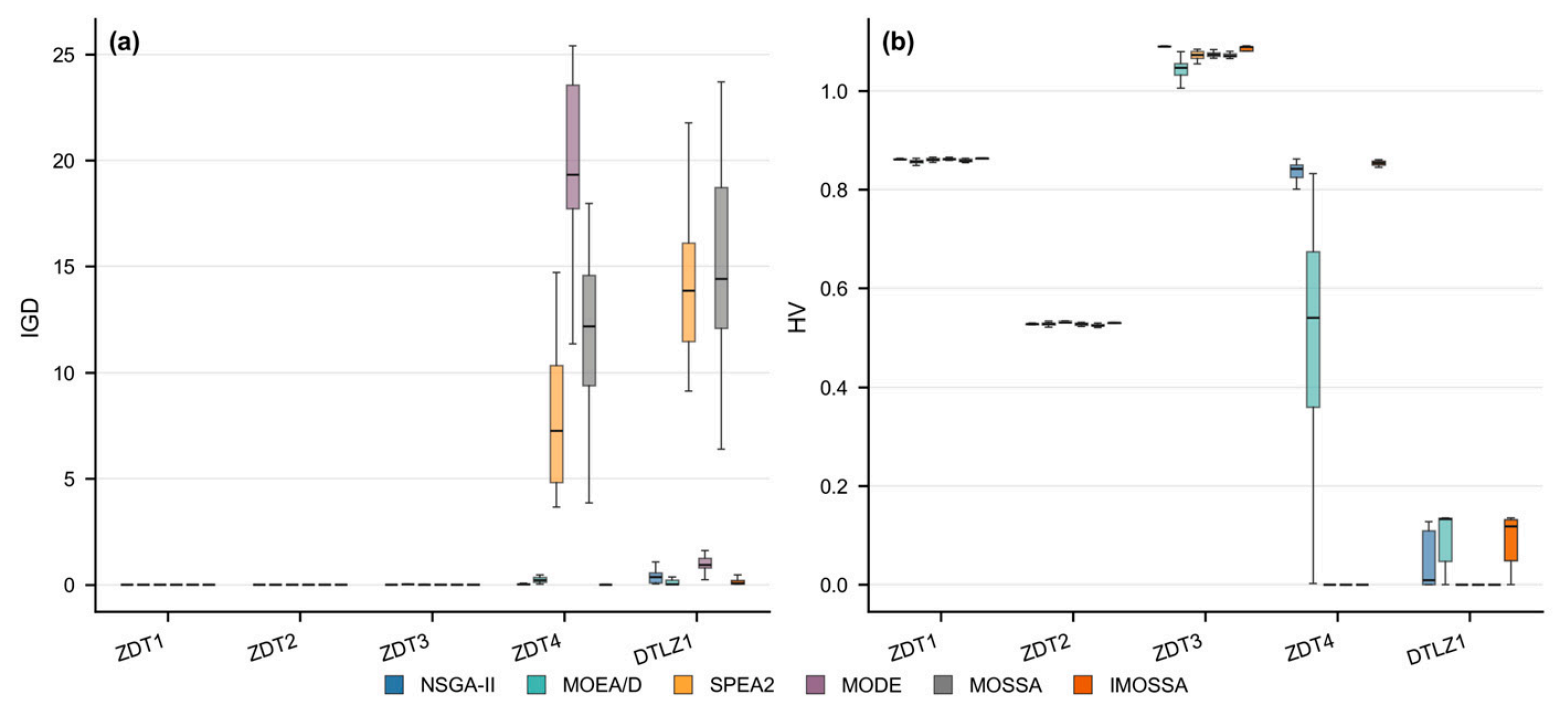
Boxplots of the performance metrics for different algorithms: (**a**) IGD; (**b**) HV. The colors correspond to the algorithms shown in the legend.

Figure 8 summarizes the statistical distributions of IGD and HV. Overall, IMOSSA performs stably on ZDT1, ZDT2, and ZDT4, with its clearest advantage observed on the multimodal ZDT4 function. A direct comparison with NSGA-II shows that IMOSSA achieves lower GD and IGD and higher HV on ZDT1, ZDT2, ZDT4, and DTLZ1, while also obtaining lower Spacing values on all five test problems. On ZDT4, in particular, IMOSSA reduces IGD from 0.0401 to 0.0195 and increases HV from 0.8304 to 0.8479 compared with NSGA-II, indicating a stronger ability to avoid multimodal local Pareto regions. However, NSGA-II performs better in terms of GD, IGD, and HV on the discontinuous ZDT3 front. Therefore, IMOSSA is not claimed to be universally superior to NSGA-II; rather, it provides a more balanced combination of convergence, solution-distribution uniformity, and late-stage search capability across the tested problems.

From the algorithmic perspective, this balanced performance is mainly attributed to three complementary mechanisms. Chaotic opposition-based elite initialization expands the initial search coverage, density-penalized archive guidance reduces repeated attraction toward locally dense archive regions, and stagnation-triggered differential–Cauchy perturbation restores search activity when archive improvement slows. Compared with MOSSA, IMOSSA achieves lower GD and Spacing values and higher HV values on ZDT1, ZDT2, and ZDT4. The improvement is most pronounced on ZDT4, where HV increases from 0 for MOSSA to 0.8479±0.0190 for IMOSSA. Although its advantage is not uniform across all metrics and front structures, the benchmark results demonstrate that the integrated strategies improve the overall reliability and diversity of the Pareto search. Accordingly, IMOSSA is regarded as a computationally reliable Pareto-search engine for the subsequent mine ventilation airflow distribution design case.

### 4.2. System-Oriented Case Study of Airflow Distribution in a Mine Ventilation Network

#### 4.2.1. System Description and Determination of Adjustable Branch Bounds

**Figure 9:**
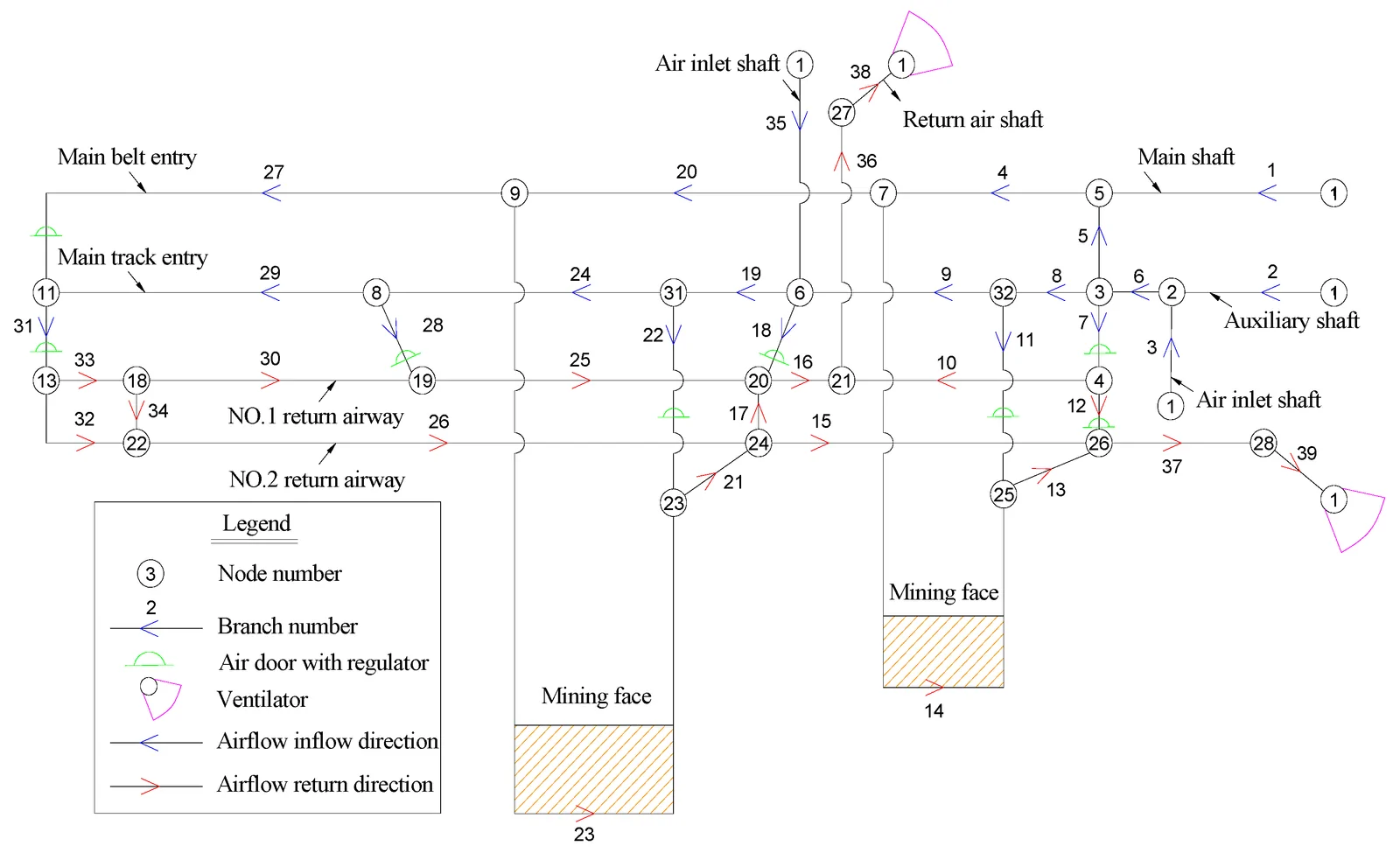
Topological structure of the mine ventilation network. Circled numbers denote node IDs, numbers adjacent to the airways denote branch IDs, and hatched regions represent mining faces. Blue and red arrows indicate intake-airflow and return-airflow directions, respectively.

To verify the engineering applicability of the proposed bi-objective airflow distribution design model, a mine ventilation network reported in [32] is used as the computational case. Its topology is shown in Figure 9. The system consists of intake shafts, auxiliary intake airways, main haulage roadways, mining and heading faces, return airways, and fan roadways. Following a graph-theoretic modeling approach, the system is abstracted as a directed ventilation network with 32 nodes and 39 branches. Nodes represent airway junctions or ventilation-facility connection points, whereas branches represent airways or fan boundaries with specified resistance and airflow direction. From a systems perspective, the case network consists of interacting nodes, airway branches, fan-boundary branches, adjustable components, and air-demand constraints, forming a coupled ventilation system with multiple feasible design states. The arrows in Figure 9 indicate the initial airflow directions, and the branch numbers correspond to the network parameters listed in Table 2.

The case data include the start and end nodes of each branch, initial branch resistance, solved initial airflow, and prescribed airflow bounds. Initial resistances are derived from the original airway resistance parameters, and the initial airflow distribution is obtained by solving the ventilation network under the original resistance condition. To ensure basic air-supply requirements, airflow constraints are specified according to branch function, initial operating condition, and ventilation design requirements. General ventilation branches are assigned allowable fluctuation intervals based on their initial airflow values; mining faces, main return airways, and key ventilation branches are assigned tighter bounds; and local high-resistance, low-flow branches retain certain adjustment flexibility to avoid over-constraining the feasible search space. Branches 38 and 39 are treated as fan-boundary branches, and their airflow bounds are set to 80–120% of the initial airflow, namely 63.2–94.8 m^3^/s for branch 38 and 73.6–110.4 m^3^/s for branch 39.

As shown in Table 2, most branches satisfy the prescribed airflow constraints under the initial condition, but several trunk and return-air branches still exhibit clear airflow redundancy. For example, branches 4, 6, 16, 19, 31, and 35 have initial airflow values substantially above their lower bounds, indicating that the original ventilation scheme has potential for reducing theoretical ventilation air power while maintaining air-supply safety. Since excessive air supply increases fan load and ineffective resistance loss, this case is suitable for evaluating the proposed multi-objective airflow distribution design method.

**Figure 10:**
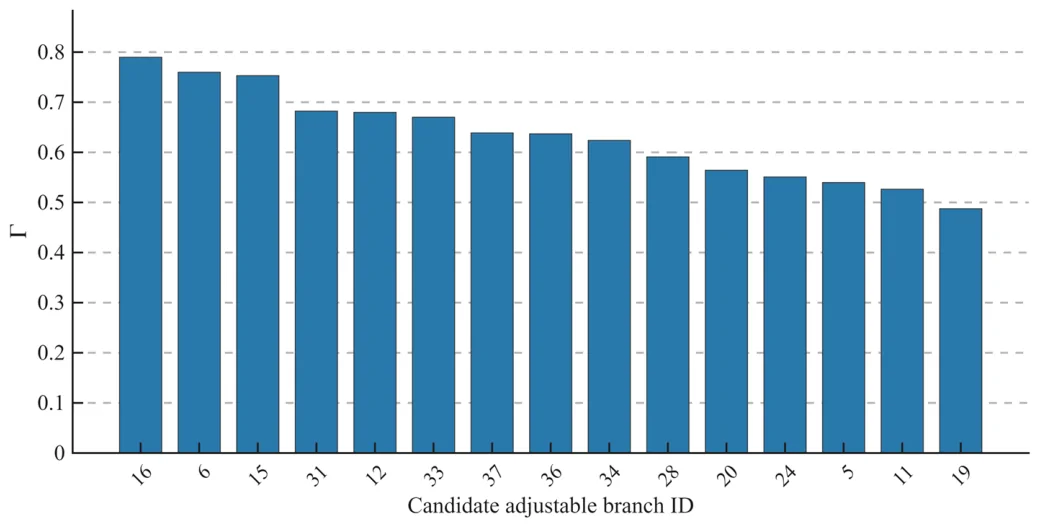
Ranking of candidate adjustable branches by comprehensive regulation coefficient.

For optimization-variable construction, the target-branch sensitivity, non-target disturbance sensitivity, and comprehensive regulation coefficient $\Gamma_{j}$ are computed from the airflow responses obtained by independently applying a ±1% resistance perturbation to each candidate branch. Candidate adjustable branches are then ranked, as shown in Figure 10. A larger $\Gamma_{j}$ indicates stronger control over demand-critical airflow and weaker disturbance to non-target regions. According to the ranking, the top 15 branches are selected as the maximum adjustable branch set: branches 16, 6, 15, 31, 12, 33, 37, 36, 34, 28, 20, 24, 5, 11, and 19. In the subsequent analyses with K=3, 5, 7,…, 15, smaller adjustable branch sets are nested subsets of larger ones, ensuring consistency and comparability among different adjustment scales.

**Figure 11:**
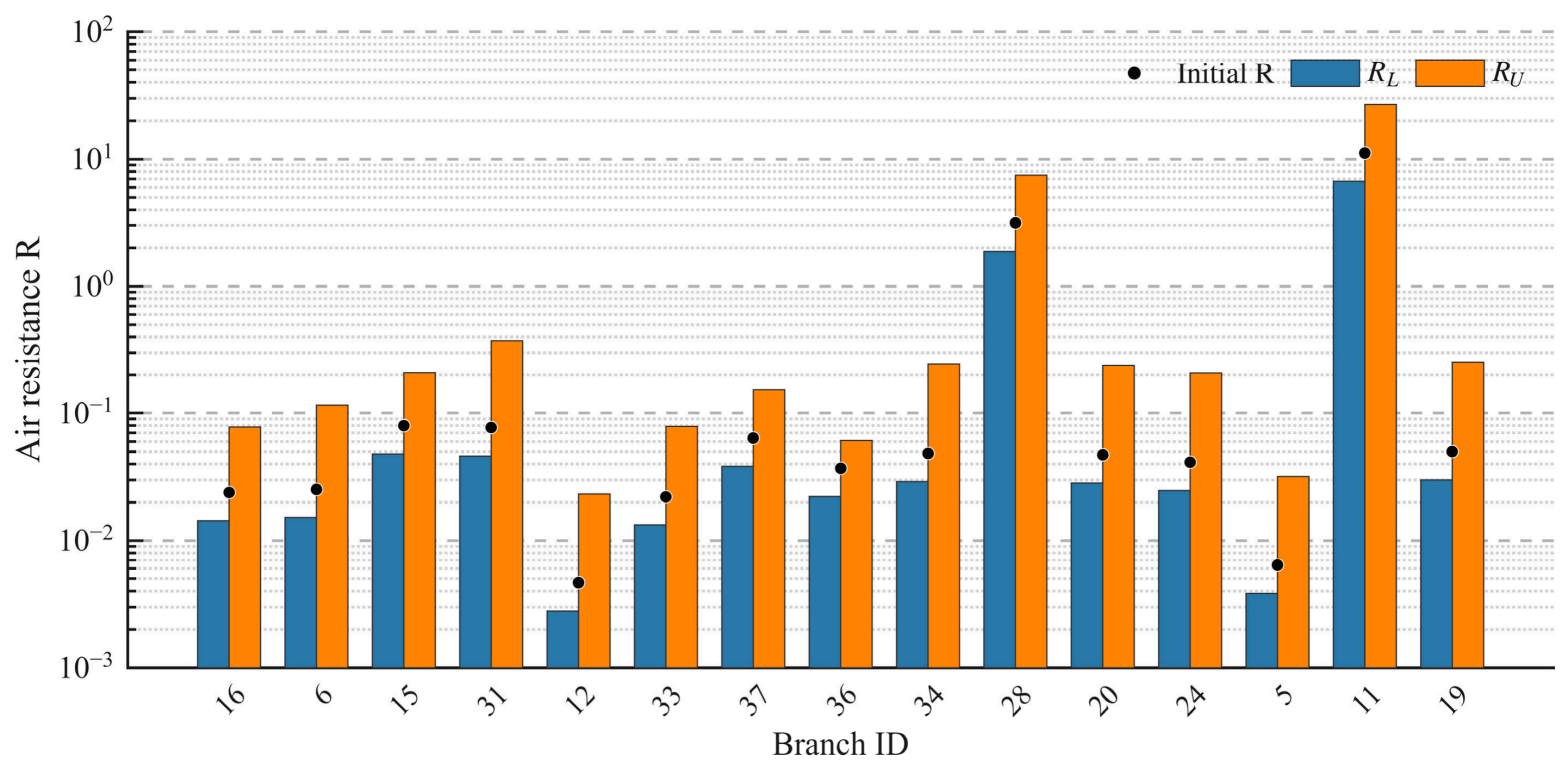
Resistance optimization bounds of the maximum candidate adjustable branch set.

After the maximum candidate set is identified, the resistance multiplier of each selected branch is scanned in both the resistance-increasing and resistance-decreasing directions. The first multiplier at which the local target-airflow sensitivity decreases to 30% of its initial value is taken as the sensitivity-derived boundary and combined with the engineering limits to determine the branch-specific resistance interval $\left[R_{j}^{L},R_{j}^{U}\right]$, as shown in Figure 11. These intervals constitute the lower and upper bounds of the corresponding decision variables in IMOSSA. Because the initial resistance and airflow response vary substantially among branches, $R_{j}^{L}\;$and $R_{j}^{U}$ show clear nonuniformity. In particular, branches 28 and 11 have much larger resistance intervals than the other branches; therefore, Figure 11 is plotted on a logarithmic scale. This result confirms that uniform proportional bounds cannot adequately represent differences in branch adjustability and engineering feasibility. The following subsection uses K=15 for multi-algorithm comparison, and Section 4.2.3 further analyzes the effect of different K values.

#### 4.2.2. Multi-Algorithm Comparison Under Different System Design Preferences

After branch screening and bound determination, NSGA-II, MOEA/D, SPEA2, MODE, MOSSA, and IMOSSA are used to solve the bi-objective airflow distribution design model. To ensure comparability, all algorithms use the same K=15 adjustable branch set, resistance bounds, airflow constraints, and ventilation network solver. The two objectives are theoretical ventilation air power $F_{1}\;$and total adjustable-branch pressure-drop variation $F_{2}$. Under the initial condition, $F_{1}\;$is 313.607 kW and $F_{2}\;$is 0 Pa.

A unified computational budget is adopted: the population size is 30, the maximum number of iterations is 300, the archive capacity is 100, and each algorithm is independently run 10 times. All candidate solutions are repaired to their bounds after updating and are then re-evaluated by the ventilation network solver. Infeasible candidates participate in the search through a constraint-violation penalty, whereas the Pareto sets, figures, and statistics reported in this paper are based on the true objective values $F_{1}\;$and $F_{2}$. Because the engineering case has no known true Pareto front, GD and IGD are not used as primary indicators; instead, feasibility, Pareto-set distribution, objective range, archive size, Spacing, and runtime are used for comparison.

**Figure 12:**
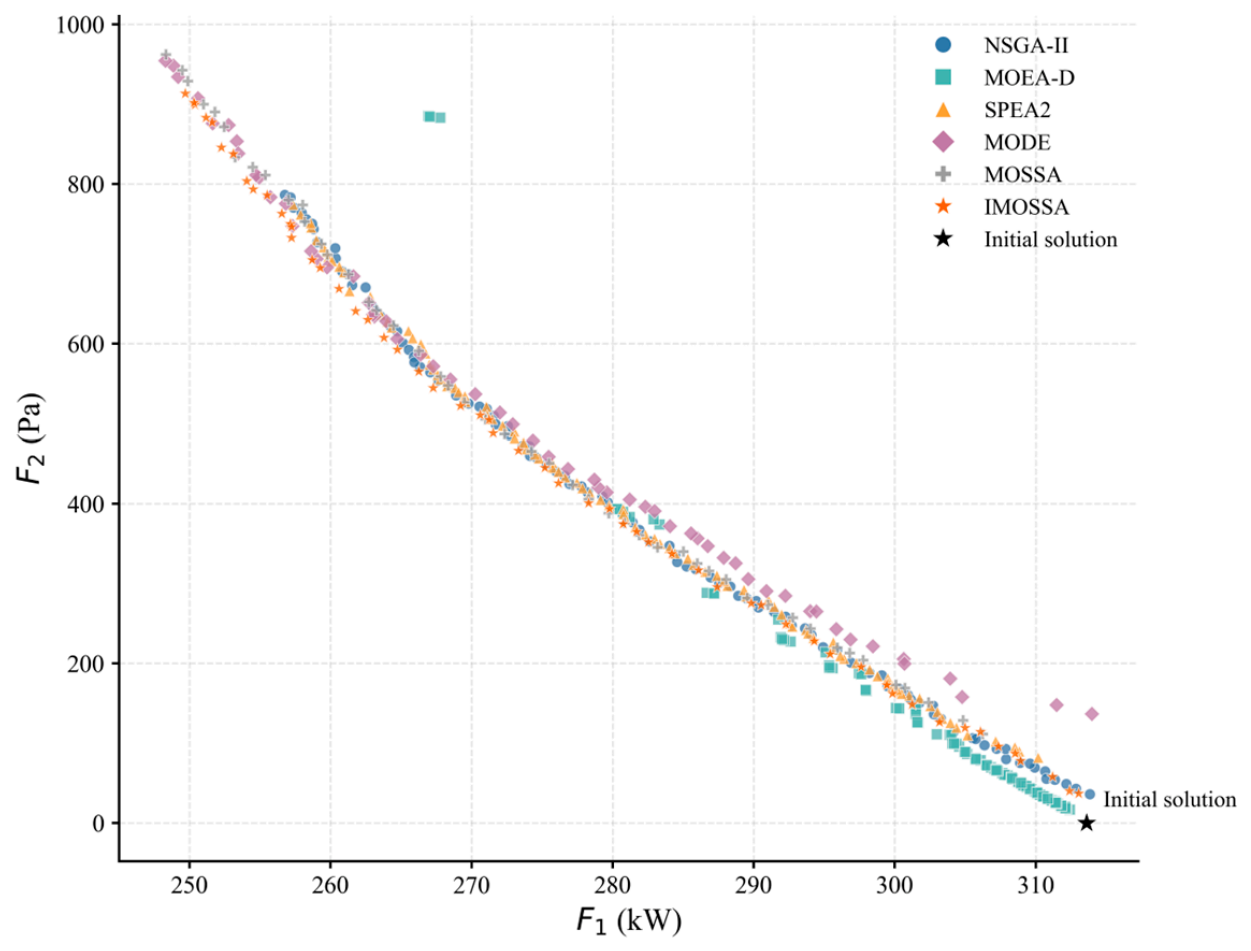
Pareto solution sets obtained by different algorithms.

Figure 12 shows the feasible Pareto solution sets obtained by the six algorithms. The initial solution lies in the high-power and zero-disturbance region, whereas the non-dominated solutions move toward lower $F_{1}$, indicating that adjustable branch settings can effectively reduce theoretical ventilation air power. Meanwhile, $F_{2}\;$generally increases as $F_{1}\;$decreases, revealing the intrinsic trade-off between air power reduction and adjustment-disturbance control. This confirms that Pareto optimization can provide candidate design schemes for different engineering preferences.

All six algorithms generate solution sets satisfying the airflow and network-balance constraints, with a feasible ratio of 1. In terms of low-power search, MODE, MOSSA, and IMOSSA perform favorably. Their minimum $F_{1}\;$values are 248.293 kW, 248.318 kW, and 249.707 kW, respectively, all about 20% lower than the initial condition. By contrast, the minimum $F_{1}\;$values of NSGA-II, SPEA2, and MOEA/D are 256.747 kW, 257.330 kW, and 266.935 kW, respectively, showing relatively limited power-reduction depth.

**Figure 13:**
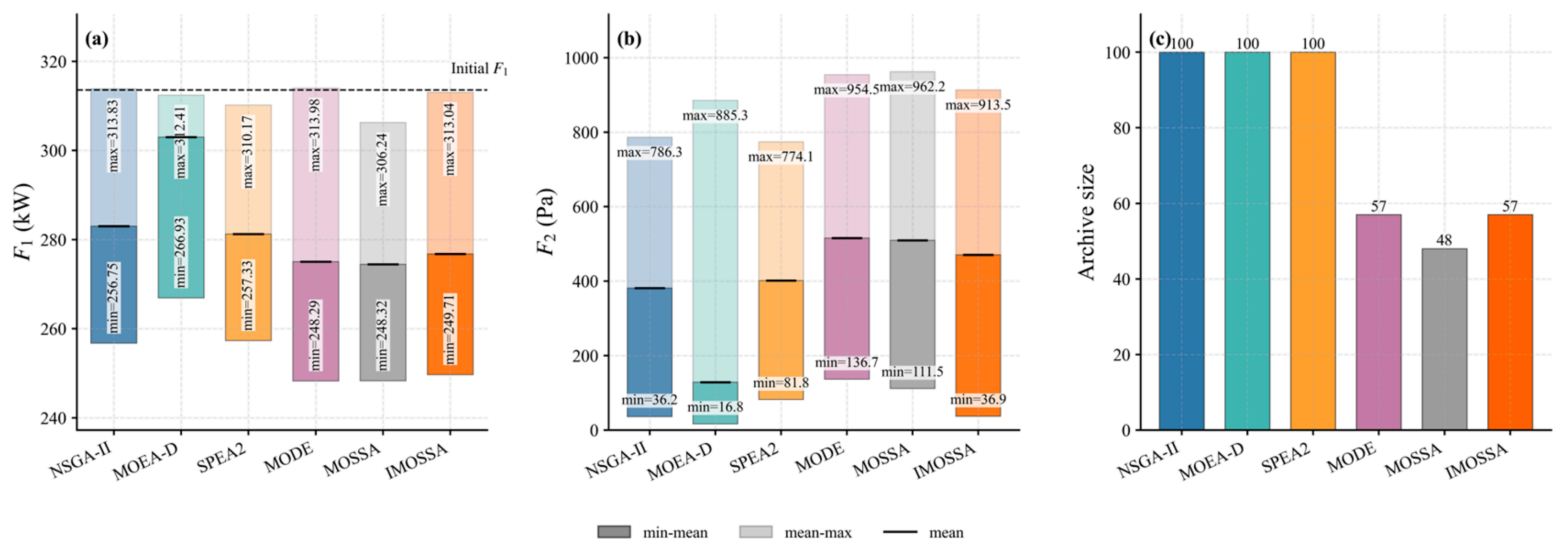
Comparison of key optimization indicators for different algorithms: (**a**) variation ranges and mean values of $F_{1}$, where the dashed horizontal line denotes the initial $F_{1}$; (**b**) variation ranges and mean values of $F_{2}$; (**c**) archive sizes.

Figure 13 further compares the key optimization indicators. MODE, MOSSA, and IMOSSA have $F_{1}\;$intervals closer to the low-power region, indicating stronger energy-saving search capability in this case. NSGA-II and SPEA2 show more concentrated $F_{1}\;$intervals, suggesting relatively stable solution distributions, whereas MOEA/D tends to retain lower-disturbance solutions. MOEA/D achieves the smallest $F_{2}\;$value of 16.784 Pa, while NSGA-II and IMOSSA also maintain relatively low minimum $F_{2}\;$values of 36.163 Pa and 36.929 Pa, respectively. The archive sizes of NSGA-II, MOEA/D, and SPEA2 all reach 100, whereas MODE, MOSSA, and IMOSSA generate 57, 48, and 57 non-dominated solutions, respectively. Combined with Figure 12, these latter algorithms concentrate more strongly in the low-power region, leading to relatively limited distribution uniformity.

Overall, the algorithms display different system design preferences. MOEA/D is suitable for preserving low-disturbance schemes but provides limited power reduction; MODE and MOSSA obtain deeper energy-saving solutions but with larger pressure-drop variation; NSGA-II and SPEA2 provide larger archive sizes and better distribution uniformity but less energy-saving depth. IMOSSA is not optimal for every single objective extreme, yet its minimum $F_{1}\;$is close to those of MODE and MOSSA, its minimum $F_{2}\;$is close to that of NSGA-II, and it shows balanced performance. Therefore, the IMOSSA Pareto set provides useful candidates for energy-priority, low-disturbance, and compromise airflow distribution design.

#### 4.2.3. Effect of Adjustable Branch Number on Design Freedom and Implementation Complexity

To analyze the influence of the number of adjustable branches on airflow optimization, the first K=3, 5, 7, 9, 11, 13, and 15 candidate branches in the sensitivity ranking are selected to form adjustable branch sets, and each corresponding model is solved using IMOSSA. Unlike the multi-algorithm comparison, the optimization algorithm, airflow constraints, fan-boundary conditions, and algorithm parameters are fixed, and only K is varied. In this section, K is not only the number of decision variables in the optimization model but also represents the number of adjustable components that may need to be implemented in the ventilation system. Therefore, increasing K simultaneously increases design freedom, potential energy-saving capability, and field implementation complexity. The initial theoretical ventilation air power is 313.607 kW, and the pressure-drop variation is 0 Pa.

**Figure 14:**
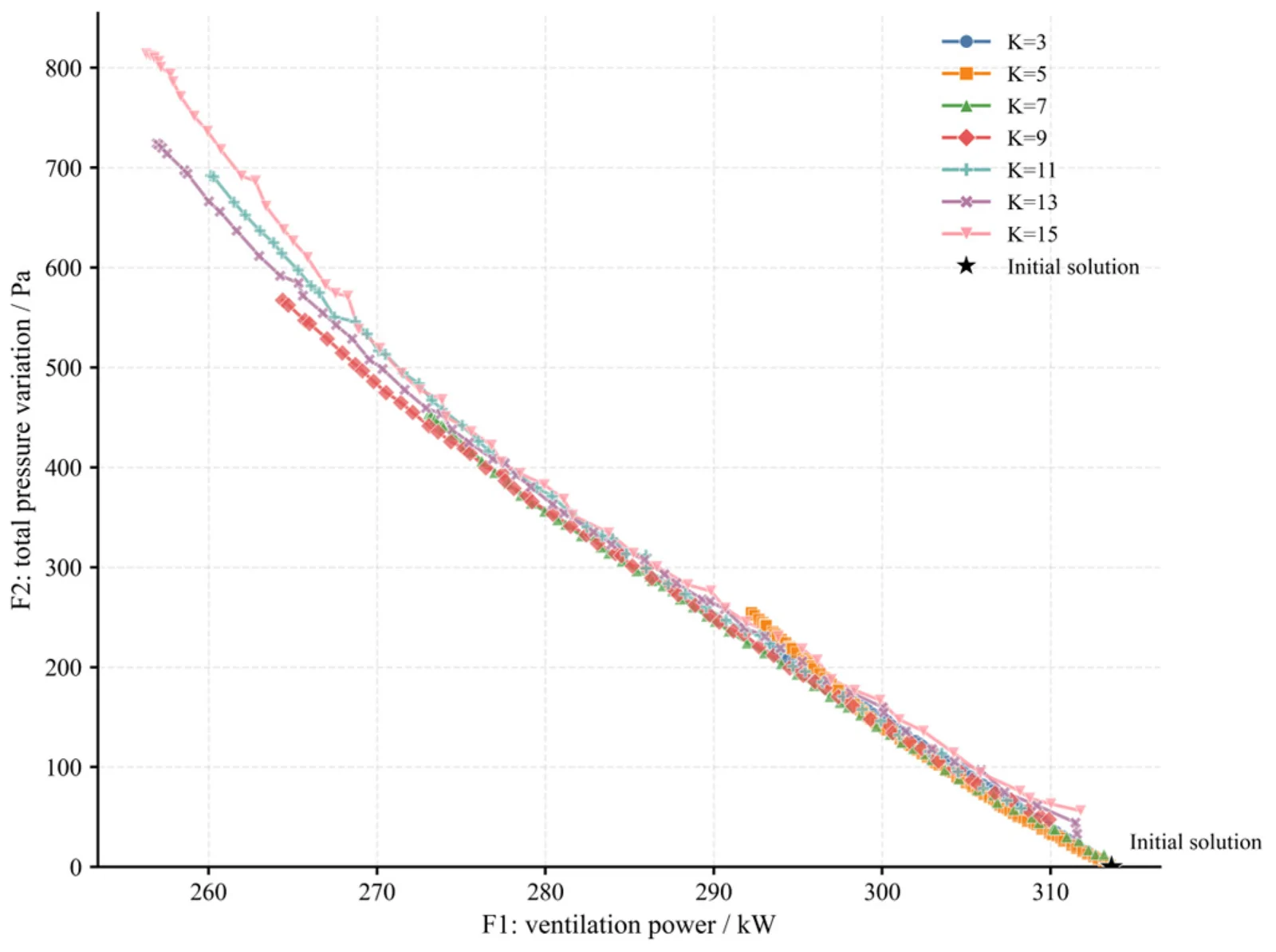
Pareto solution sets for different numbers of adjustable branches (K=3, 5, 7, 9, 11, 13, and 15); the black star denotes the initial solution.

Figure 14 shows the Pareto solution sets under different K values. When K=3 or K=5, the solutions are concentrated in the high-power, low-disturbance region, indicating that a small number of highly sensitive branches can preserve a small adjustment magnitude but cannot fully reduce air power. As K increases to 7 and 9, the Pareto front expands markedly toward lower air power, implying that additional adjustable branches improve the ability of the network to redistribute airflow. When K≥11, the fronts become closer, suggesting diminishing marginal benefit from further increasing the number of adjustable branches.

**Figure 15:**
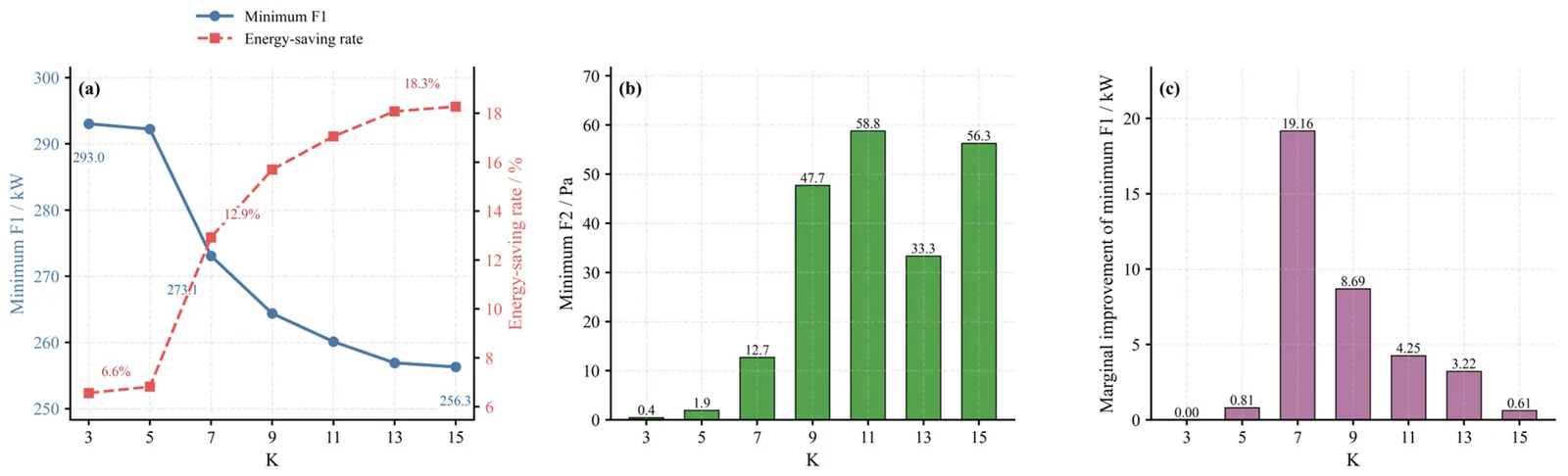
Optimization indicators under different numbers of adjustable branches: (**a**) minimum air power and energy-saving rate; (**b**) minimum pressure-drop variation; (**c**) marginal improvement between adjacent adjustment scales.

Figure 15 and Table 3 summarize the minimum air power, minimum pressure-drop variation, and marginal improvement. At K=3, the minimum $F_{1}\;$is 293.033 kW, 6.56% lower than the initial condition. At K=5, it decreases only to 292.228 kW, with a marginal improvement of 0.805 kW, indicating limited independent regulation by the first five branches. The largest improvement occurs from K=5 to K=7, where $F_{1}\;$decreases from 292.228 kW to 273.069 kW, and the marginal improvement reaches 19.159 kW. Thereafter, $F_{1}$ continues to decline, but the marginal improvement gradually decreases. From K=13 to K=15, $F_{1}\;$decreases only from 256.909 kW to 256.298 kW, with an additional benefit of 0.611 kW.

The minimum $F_{2}\;$is not strictly monotonic with K. For K=3 and K=5, the minimum pressure-drop variations are 0.441 Pa and 1.929 Pa, respectively, showing that a small adjustment set has limited impact on the initial pressure-drop distribution. Deeper energy saving generally requires more pronounced local pressure-drop redistribution. Notably, the minimum $F_{2}\;$at K=13 is 33.330 Pa, lower than those at K=11 and K=15, indicating that increasing the number of adjustable branches does not necessarily increase disturbance; the result also depends on branch location, resistance bounds, and Pareto-set distribution.

Different K values therefore correspond to different engineering scenarios and system design complexities. K=3 and K=5 are suitable for conservative low-disturbance adjustment but provide limited energy saving. K=7 to K=11 represents the rapid improvement stage. K=13 maintains a low air power level with relatively low pressure-drop disturbance and provides a favorable engineering trade-off in this case. K=15 gives the lowest power, but its improvement over K=13 is small. This result reflects a typical system design trade-off: increasing the number of adjustable components improves the flexibility of airflow redistribution, but the marginal energy-saving benefit decreases while the implementation complexity increases. A complete economic assessment would require mine-specific data on regulator installation, construction, and maintenance costs; therefore, K=13 is not claimed to be economically optimal. It is selected as a representative engineering trade-off for this case because, compared with K=15, it requires two fewer adjustable locations while sacrificing only 0.611 kW in minimum air power reduction. In addition, the minimum $F_{2}$ of the K=13 Pareto set is 33.330 Pa, lower than the 56.255 Pa obtained at K=15. Accordingly, K=15 is retained only as the energy-saving-limit comparison.

It should be noted that the runtime does not vary monotonically with the number of adjustable branches. Under the same population size, iteration number, and objective-evaluation budget, the total runtime is determined not only by the decision dimension but also by the convergence behavior of the inner nonlinear ventilation network solver. Different resistance configurations may result in different network-solution iteration counts and function evaluations; therefore, some configurations with a smaller K may require a longer runtime than those with a larger K.

#### 4.2.4. Pareto-Based Decision Support for Representative Airflow Distribution Schemes

The output of multi-objective airflow optimization is not a single optimum but a Pareto set representing trade-offs between theoretical ventilation air power and adjustment disturbance. Different schemes correspond to different engineering preferences. Low-power schemes generally require larger local resistance adjustment, whereas low-disturbance schemes remain close to the initial state but yield limited energy saving. Based on the preceding K-comparison, the Pareto set at K=13, containing 53 feasible non-dominated solutions, is selected for representative-scheme analysis.

Three representative schemes are selected: the energy-priority scheme $S_{1}$, the low-disturbance scheme $S_{2}$, and the compromise scheme $S_{3}$. $S_{1}\;$is the non-dominated solution with the minimum $F_{1}$, representing the maximum energy-saving potential. $S_{2}\;$is the solution with the minimum $F_{2}$, representing the lowest engineering disturbance. $S_{3}$ is selected using the normalized ideal-point distance method: $F_{1}$ and $F_{2}$ are range-normalized over the Pareto set, and the scheme closest to the ideal point $\left(0,0\right)$ is selected. The $F_{1,min}$ and $F_{2,min}$ statistics in Table 3 generally correspond to different Pareto solutions and should not be interpreted as simultaneous extremes of one scheme. The three representative schemes can also be interpreted as three system design strategies: $S_{1}$ represents an energy-priority system design, $S_{2}$ represents a low-disturbance system design, and $S_{3}$ represents a balanced system design.

**Figure 16:**
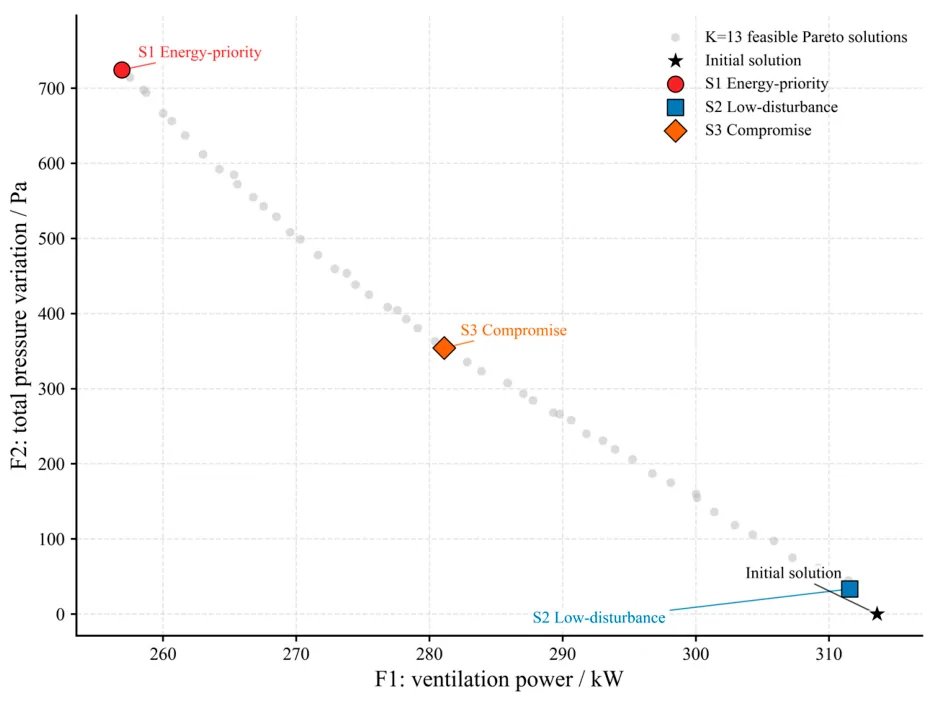
Positions of representative Pareto schemes in the non-dominated solution set.

Figure 16 shows the positions of the three schemes on the K=13 Pareto front. $S_{1}$ lies at the low-$F_{1}$ end, with the lowest air power but the largest pressure-drop variation, implying strong local pressure redistribution. $S_{2}$ lies near the low-$F_{2}$ end and remains close to the initial pressure-drop distribution, indicating minimal disturbance but limited power reduction. $S_{3}$ lies in the middle of the front and provides a balanced compromise between air power reduction and pressure-drop disturbance.

**Figure 17:**
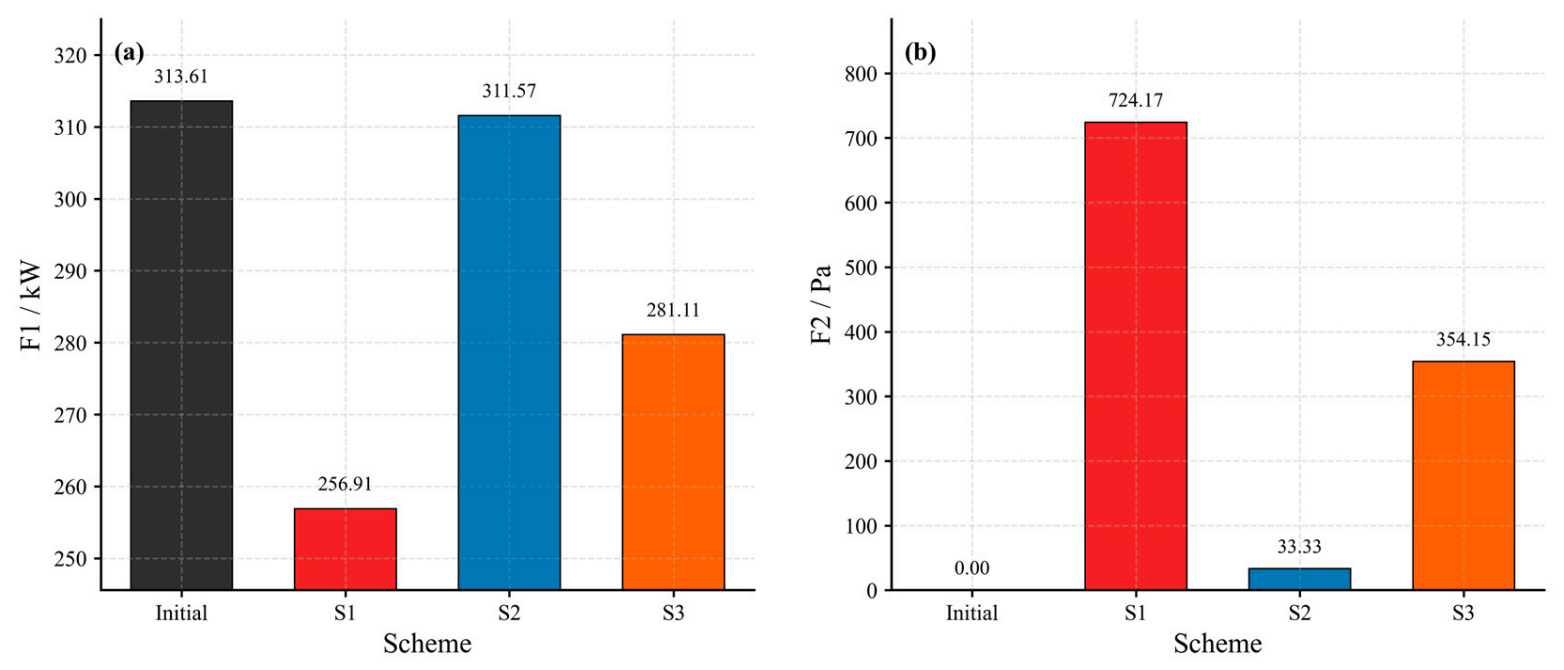
Objective comparison of representative Pareto schemes: (**a**) comparison of $F_{1}$ for the Initial, $S_{1}$, $S_{2}$, and $S_{3}$ schemes; (**b**) comparison of $F_{2}$ for the Initial, $S_{1}$, $S_{2}$, and $S_{3}$ schemes.

As shown in Figure 17 and Table 4, the initial air power is 313.61 kW. The energy-priority scheme $S_{1}$ reduces air power to 256.91 kW, corresponding to an 18.08% reduction, but its $F_{2}$ reaches 724.17 Pa, indicating high implementation intensity. The low-disturbance scheme $S_{2}$ has $F_{2}=33.33$ Pa, causing the least change to the initial ventilation state, but its air power remains 311.57 kW and its energy-saving rate is only 0.65%. The compromise scheme $S_{3}$ reduces air power to 281.11 kW, corresponding to a 10.36% reduction, while maintaining $F_{2}$ at 354.15 Pa, substantially lower than that of $S_{1}$.

From a decision-support perspective, the three schemes have distinct application boundaries. If a mine is undergoing design optimization or energy-saving retrofit and larger local resistance adjustments are acceptable, $S_{1}$ can be used to exploit the maximum energy-saving potential. If the existing ventilation system is stable and only small corrective adjustments are desired, $S_{2}$ is more conservative. If both power reduction and adjustment intensity must be considered, $S_{3}$ is recommended as the compromise design. Thus, the Pareto set provides not only a low-power limit scheme but also low-disturbance and balanced alternatives, transforming the numerical optimization output into a decision-support tool for differentiated mine ventilation system design.

## 5. Discussion

This study treats mine ventilation airflow distribution as a system-oriented multi-objective design problem for coupled ventilation networks. Under a prescribed network topology, fan-boundary conditions, and demand-critical airflow constraints, the proposed framework generates multiple comparable airflow distribution schemes. By treating demand-critical airflow as constraints and using theoretical ventilation air power and adjustable-branch pressure-drop deviation as two separate system objectives, the model preserves the physical meaning of different quantities and improves the interpretability of Pareto schemes for ventilation system design.

Sensitivity-based branch screening and differentiated resistance bounds are important for reducing system design complexity. In a mine ventilation network, different branches have substantially different influences on demand-critical airflow. If all branch resistances are used as design variables, the decision space expands rapidly, and many low-contribution variables may be introduced. The proposed resistance-perturbation analysis identifies high-impact adjustable branches, while sensitivity attenuation defines branch-specific search bounds and forms a compact decision domain. The comparison among different numbers of adjustable branches shows that increasing the adjustable component scale improves the flexibility of airflow redistribution, but the energy-saving benefit exhibits marginal reduction; therefore, the number of adjustable branches should be determined by balancing system performance, pressure-drop disturbance, and implementation complexity.

The Pareto solution set provides a useful decision-support representation for different ventilation design preferences. The benchmark results indicate that, relative to MOSSA, IMOSSA achieves a more balanced combination of initial population coverage, archive diversity maintenance, and late-stage escape capability. This advantage is particularly evident in constrained and multimodal search spaces, although it is not reflected uniformly across all benchmark metrics. The engineering case further demonstrates its applicability to the coupled optimization problem involving network solving, airflow constraints, and continuously adjustable branch settings. The representative Pareto schemes show that low-power, low-disturbance, and compromise solutions correspond to different system design strategies. In practical use, the optimization can be performed offline during ventilation system design or retrofit analysis, and the resulting Pareto set can be stored as a candidate scheme library for engineering selection. Therefore, the computational cost of the meta-heuristic search does not prevent its application to the design-stage airflow distribution problem.

The present study is based on a steady-state ventilation network case, and the objective function uses fan-boundary air power to represent theoretical ventilation air power. In practical applications, fan performance curves, natural ventilation pressure, seasonal operating conditions, and discrete regulation facilities may further affect the final design scheme. Future work may extend the current framework to dynamic ventilation demand, multi-condition system behavior, discrete regulation components, and robust system optimization to improve its applicability to practical mine ventilation system design. For computational acceleration, future work may dynamically update the minimum-impact tree and the associated loop basis after resistance changes. The spanning tree from the initial or preceding resistance state can be used as a warm-start tree and updated through local edge replacement, thereby avoiding complete reconstruction and reducing the convergence cost of the inner network solver.

## 6. Conclusions

This study proposed a biomimetic multi-objective optimization framework for design-stage airflow distribution in mine ventilation systems based on sensitivity screening and an improved multi-objective sparrow search algorithm (IMOSSA). Adjustable branch settings are used as design variables; demand-critical branch airflow, fan-boundary airflow, and resistance bounds are imposed as constraints; and theoretical ventilation air power and adjustable-branch pressure-drop deviation are minimized as two conflicting objectives. The main conclusions are as follows.

(1) A bi-objective airflow distribution design model was established for mine ventilation systems. The model integrates network balance, demand-critical airflow, fan-boundary conditions, and adjustable-resistance constraints into a unified optimization framework. By separating airflow requirements from the objective functions, the model avoids mixing heterogeneous physical quantities in a single weighted fitness function and improves the interpretability of Pareto design schemes.
(2) A sensitivity-based decision-domain construction strategy was developed. Resistance-perturbation response and sensitivity attenuation are used to screen high-impact adjustable branches and construct branch-specific search intervals, thereby forming a compact and physically feasible decision domain. The comparison among different adjustable branch numbers shows that K=13 provides a reasonable balance between energy-saving performance and implementation complexity. When K increases from 13 to 15, the minimum theoretical ventilation air power decreases only from 256.909 kW to 256.298 kW, with a marginal improvement of only 0.611 kW, indicating that blindly increasing the number of adjustable branches is unnecessary.
(3) IMOSSA was used as a biomimetic Pareto-search engine inspired by sparrow foraging and vigilance behaviors. By integrating chaotic opposition-based elite initialization, density-penalized external-archive guidance, and stagnation-triggered differential–Cauchy perturbation, IMOSSA improves initial search coverage, Pareto-front diversity, and late-stage escape capability. Benchmark tests show that IMOSSA has competitive convergence and distribution performance; in particular, on the multimodal ZDT4 function, its mean GD, IGD, and HV values reach 0.0096, 0.0195, and 0.8479, respectively. These results verify the computational reliability of the proposed biomimetic optimizer for subsequent ventilation system optimization.
(4) The mine ventilation network case demonstrates that the proposed framework can generate feasible Pareto airflow distribution schemes satisfying system constraints. For K=13, the compromise scheme reduces the model-computed ventilation air power from 313.61 kW to 281.11 kW, corresponding to a reduction of 10.36%, while maintaining the pressure-drop deviation at 354.15 Pa. The energy-priority scheme further reduces the theoretical ventilation air power to 256.91 kW, corresponding to a reduction of 18.08%, but with a larger pressure-drop deviation. These results show that the Pareto set can support energy-priority, low-disturbance, and balanced ventilation design decisions.

In summary, the proposed biomimetic multi-objective optimization framework provides a computable and interpretable decision-support method for airflow distribution design, adjustable branch setting, and multi-scheme selection in mine ventilation systems. Future research may incorporate dynamic ventilation demand, multi-condition system behavior, fan operating characteristics, discrete regulation components, and robust optimization mechanisms to enhance its applicability to practical ventilation design scenarios.

## Figures and Tables

**Table 1:** Performance comparison of different algorithms on benchmark functions.

Function	Metric	NSGA-II	MOEA/D	SPEA2	MODE	MOSSA	IMOSSA
ZDT1	GD	0.0037 ± 0.0010	0.0109 ± 0.0028	0.0070 ± 0.0021	0.0064 ± 9.59 × 10^−4^	0.0081 ± 0.0017	0.0033 ± 6.61 × 10^−4^
ZDT1	IGD	0.0098 ± 5.22 × 10^−4^	0.0110 ± 0.0026	0.0088 ± 0.0016	0.0080 ± 8.59 × 10^−4^	0.0098 ± 0.0015	0.0086 ± 2.97 × 10^−4^
ZDT1	HV	0.8620 ± 8.40 × 10^−4^	0.8564 ± 0.0045	0.8610 ± 0.0030	0.8623 ± 0.0016	0.8592 ± 0.0028	0.8635 ± 6.45 × 10^−4^
ZDT1	Spacing	0.0146 ± 0.0017	0.0077 ± 0.0022	0.0082 ± 5.39 × 10^−4^	0.0068 ± 4.66 × 10^−4^	0.0084 ± 8.83 × 10^−4^	0.0069 ± 0.0012
ZDT2	GD	0.0037 ± 0.0010	0.0080 ± 0.0017	0.0033 ± 0.0012	0.0065 ± 0.0014	0.0063 ± 0.0025	0.0024 ± 6.55 × 10^−4^
ZDT2	IGD	0.0100 ± 4.98 × 10^−4^	0.0075 ± 0.0016	0.0366 ± 0.1350	0.0081 ± 0.0012	0.0691 ± 0.1851	0.0082 ± 2.36 × 10^−4^
ZDT2	HV	0.5288 ± 9.99 × 10^−4^	0.5286 ± 0.0031	0.5115 ± 0.0945	0.5283 ± 0.0025	0.4850 ± 0.1283	0.5309 ± 7.21 × 10^−4^
ZDT2	Spacing	0.0141 ± 0.0017	0.0046 ± 7.58 × 10^−4^	0.0084 ± 8.53 × 10^−4^	0.0072 ± 7.78 × 10^−4^	0.0081 ± 0.0011	0.0066 ± 9.59 × 10^−4^
ZDT3	GD	0.0026 ± 9.62 × 10^−4^	0.0161 ± 0.0090	0.0077 ± 0.0026	0.0081 ± 0.0023	0.0075 ± 0.0015	0.0059 ± 0.0141
ZDT3	IGD	0.0122 ± 0.0058	0.0253 ± 0.0109	0.0113 ± 0.0031	0.0112 ± 0.0025	0.0110 ± 0.0016	0.0163 ± 0.0112
ZDT3	HV	1.0892 ± 0.0068	1.0428 ± 0.0233	1.0726 ± 0.0093	1.0735 ± 0.0069	1.0729 ± 0.0050	1.0805 ± 0.0176
ZDT3	Spacing	0.0177 ± 0.0025	0.0151 ± 0.0034	0.0106 ± 6.78 × 10^−4^	0.0090 ± 7.10 × 10^−4^	0.0105 ± 9.76 × 10^−4^	0.0085 ± 0.0012
ZDT4	GD	0.0108 ± 0.0041	0.4838 ± 0.6128	9.4342 ± 5.8826	23.9372 ± 4.4630	13.3282 ± 6.3269	0.0096 ± 0.0048
ZDT4	IGD	0.0401 ± 0.0442	0.2802 ± 0.2301	8.8416 ± 5.7225	19.9168 ± 3.4902	12.8220 ± 6.4818	0.0195 ± 0.0216
ZDT4	HV	0.8304 ± 0.0319	0.5016 ± 0.2313	0.0000 ± 0.0000	0.0000 ± 0.0000	0.0000 ± 0.0000	0.8479 ± 0.0190
ZDT4	Spacing	0.0131 ± 0.0023	0.1009 ± 0.2922	0.0170 ± 0.0112	0.8504 ± 0.7545	0.0161 ± 0.0089	0.0069 ± 0.0015
DTLZ1	GD	7.4301 ± 9.6979	1.0915 ± 1.2622	28.3544 ± 3.7961	2.4289 ± 1.0466	30.1265 ± 3.6578	3.3974 ± 5.8383
DTLZ1	IGD	0.3846 ± 0.3116	0.1387 ± 0.1958	13.6277 ± 4.0268	1.0066 ± 0.4536	14.9223 ± 4.8356	0.1581 ± 0.1690
DTLZ1	HV	0.0459 ± 0.0550	0.1004 ± 0.0541	0.0000 ± 0.0000	0.0087 ± 0.0307	0.0000 ± 0.0000	0.0897 ± 0.0519
DTLZ1	Spacing	3.8430 ± 5.7728	0.9106 ± 1.2771	3.0706 ± 0.9617	0.4656 ± 0.1797	3.3849 ± 0.6298	2.2929 ± 4.1382

**Table 2:** Branch parameters and airflow constraints of the mine ventilation network.

Branch	Starting Node	End Node	Resistance	Calculated Airflow	Lower Limit	Upper Limit
1	1	5	0.1866	38.18	26.73	49.63
2	1	2	0.04471	46.28	32.4	60.16
3	1	2	0.08281	34.01	23.81	44.21
4	5	7	0.02175	83.8	58.66	108.94
5	3	5	0.00639	45.62	31.93	59.31
6	2	3	0.02529	80.29	56.2	104.38
7	3	4	24.2	6.43	1.93	12.86
8	3	32	0.0051	28.23	19.76	36.7
9	32	6	0.0693	19.35	13.54	25.16
10	4	21	0.59984	1.76	0.53	3.52
11	32	25	11.18	8.89	2.67	17.78
12	4	26	0.00466	4.67	1.4	9.34
13	25	26	0.06501	41.99	37.79	50.39
14	7	25	0.65803	33.1	29.79	39.72
15	24	26	0.07999	45.34	31.74	58.94
16	20	21	0.02395	77.24	54.07	100.41
17	24	20	0.04253	23.39	16.37	30.41
18	6	20	8.21455	10.06	3.02	20.12
19	6	31	0.0501	61.82	43.27	80.37
20	7	9	0.04728	50.7	35.49	65.91
21	23	24	0.1101	39.9	35.91	47.88
22	31	23	7.04802	9.21	2.76	18.42
23	9	23	0.56521	30.69	27.62	36.83
24	31	8	0.04139	52.61	36.83	68.39
25	19	20	0.02215	43.79	30.65	56.93
26	22	24	0.14658	28.83	20.18	37.48
27	9	11	0.29988	20.01	18.01	24.01
28	8	19	3.12464	12.42	3.73	24.84
29	8	11	0.04438	40.18	28.13	52.23
30	18	19	0.10513	31.37	21.96	40.78
31	11	13	0.07703	60.2	42.14	78.26
32	13	22	0.04699	24.72	17.3	32.14
33	13	18	0.02218	35.47	24.83	46.11
34	18	22	0.04853	4.11	1.23	8.22
35	1	6	0.10465	52.53	36.77	68.29
36	21	27	0.03712	79	63.2	94.8
37	26	28	0.06391	92	73.6	110.4
38	27	1	0.01896	79	63.2	94.8
39	28	1	0.01924	92	73.6	110.4

**Table 3:** IMOSSA results under different numbers of adjustable branches.

K	Archive Size	Feasible Ratio	$F_{1,min}$/kW	Energy-Saving Rate/%	$F_{2,min}$/Pa	Marginal Improvement/kW	Runtime/s
3	95	1	293.033	6.56	0.441	0	124.85
5	95	1	292.228	6.82	1.929	0.805	476.57
7	69	1	273.069	12.93	12.695	19.159	258.16
9	66	1	264.381	15.70	47.698	8.688	204.87
11	55	1	260.126	17.05	58.759	4.255	204.67
13	53	1	256.909	18.08	33.330	3.217	406.17
15	55	1	256.298	18.27	56.255	0.611	308.58

**Table 4:** Objective comparison of representative Pareto schemes.

Scheme	Selection Rule	$F_{1}$/kW	Energy-Saving Rate/%	$F_{2}$/Pa	Constraint Status
Initial	Initial network solution	313.61	0	0	Feasible
S1	Minimum F1	256.91	18.08	724.17	Feasible
S2	Minimum F2	311.57	0.65	33.33	Feasible
S3	Minimum normalized ideal-point distance	281.11	10.36	354.15	Feasible

## Data Availability

All data generated or analyzed during this study are included in this article.
